# A gene that underwent adaptive evolution, *LAC2* (LACCASE), in *Populus euphratica* improves drought tolerance by improving water transport capacity

**DOI:** 10.1038/s41438-021-00518-x

**Published:** 2021-04-01

**Authors:** Zhimin Niu, Guiting Li, Hongyin Hu, Jiaojiao Lv, Qiwei Zheng, Jianquan Liu, Dongshi Wan

**Affiliations:** 1 State Key Laboratory of Grassland Agro-Ecosystem, School of Life Sciences, Lanzhou University, Lanzhou, Gansu People’s Republic of China; 2 Laboratory of Cell Biology, College of Life Science and Technology, Huazhong Agricultural University, Wuhan, People’s Republic of China

**Keywords:** Drought, Transgenic plants

## Abstract

Drought severely limits plant development and growth; accordingly, plants have evolved strategies to prevent water loss and adapt to water deficit conditions. However, experimental cases that corroborate these evolutionary processes are limited. The LACCASEs (*LACs*) family is involved in various plant development and growth processes. Here, we performed an evolutionary analysis of *LACs* from *Populus euphratica* and characterized the functions of *LACs* in *Arabidopsis* and poplar. The results showed that in *PeuLACs*, multiple gene duplications led to apparent functional redundancy as the result of various selective pressures. Among them, *PeuLAC2* underwent strong positive selection. Heterologous expression analyses showed that the overexpression of *PeuLAC2* alters the xylem structure of plants, including thickening the secondary cell wall (SCW) and increasing the fiber cell length and stem tensile strength. Altogether, these changes improve the water transport capacity of plants. The analysis of the physiological experimental results showed that *PeuLAC2*-OE lines exhibited a stronger antioxidant response and greater drought tolerance than WT. Three genes screened by transcriptome analysis, *NAC025*, *BG1*, and *UGT*, that are associated with SCW synthesis and drought stress were all upregulated in the *PeuLAC2*-OE lines, implying that the overexpression of *PeuLAC2* thickened the SCW, improved the water transport capacity of the plant, and further enhanced its drought tolerance. Our study highlights that genes that have undergone adaptive evolution may participate in the development of adaptive traits in *P. euphratica* and that *PeuLAC2* could be a candidate gene for molecular genetic breeding in trees.

## Highlights

- A *LAC2* gene in *P. euphratica* underwent positive selection;
- *PeuLAC2*-OE thickens the secondary cell wall;
- *PeuLAC2*-OE improves drought tolerance in plants by enhancing their water transport capacity.

## Introduction

Water is one of the foremost limiting factors for normal plant survival. Plants, especially those distributed in arid and semiarid regions, have evolved various strategies to prevent water loss or adapt to growth in conditions with water deficiency^1^. For example, at the physiological level, root hydrotropic growth is stronger in desert poplar than in other poplar plants, and its exhibits high antioxidant enzyme activities^2^. At the morphological level, water is stored in the large parenchymal cells in the swollen stems and leaves of succulent cacti. The closure or opening of stomata regulated by phytohormones^3^, together with the water transport capacity of stems (xylem pressure), promotes water balance in plants^4^. In woody plants, several studies have shown that tolerance to drought is strongly related to the structure of xylem, which is associated with the water transport capacity of plants^5^. However, experimental cases supporting the related evolutionary processes, especially those concerning the molecular regulatory mechanisms of desert poplar, remain limited.

LACCASEs (*LACs*) encode multicopper oxidases that can catalyze the oxidation of various substrates and reduce molecular oxygen (O_2_) to water (H_2_O)^6^. *LACs* have been found widely in fungi, bacteria, insects, and plants^7,8^. In plants, *LACs* are primarily involved in monomer polymerization to form phenolic biopolymers^9,10^. Increasing studies have found that *LACs* may participate in lignin synthesis and metabolism^11,12^. For example, in *Arabidopsis*, a total of 17 *LAC* members have been identified^12^, four of which (*LAC4*, *LAC11*, *LAC15*, and *LAC17*) are involved in lignin biosynthesis and regulating cell wall structure^11,13,14^. *LAC1* from *Pyrus bretschneideri* has been interfamily-transferred into *Arabidopsis*, which induced a significant increase in the lignin content and cell wall thickness of interfascicular fibers and xylem cells^15^. In *Populus trichocarpa*, *LAC3* is involved in the structural formation of normal cell walls and enhances the integrity of xylem fibers^16^. The expression of *LACs* can also be regulated by microRNAs; for example, Ptr-miR397a affects lignin content by negatively regulating *LAC* genes in *P. trichocarpa*^17^, while miR397b modulates a *LAC*, which results in both increased lignin content and seed number in *Arabidopsis*^18^. In rice, microRNA-directed LACCASE gene silencing alters lignification and contributes to the domestication of cultivated indica rice^19^. Lignification in plants is often associated with improved tolerance to biotic stresses. For example, the overexpression of *GhLAC1* from *Gossypium hirsutum* enhances lignification and leads to increased tolerance to biotic stresses, such as cotton bollworms, cotton aphids, and fungal pathogens^20^. The overexpression of *GhLAC15* enhances resistance to Verticillium wilt by increasing lignification and lignin content in plant cell walls^21^. Moreover, *LACs* also participate in the plant response to abiotic stresses^12^. In *Arabidopsis*, *Atlac2* plants exhibit reduced root elongation under dehydration conditions, while *Atlac8* plants flower earlier than wild-type plants^22^. *LACs* may also be involved in plant growth through the regulation of the flavonoid pathway. For example, *TRANSPARENT TESTA10*, a *LAC*-like enzyme, is related to the oxidative polymerization of flavonoids in the *Arabidopsis* seed coat^9^. Rice *OsChI1*, a putative *LAC* precursor that is overexpressed, increases tolerance to salt and drought stresses in *Arabidopsis*^23^. *OsLAC10* can reduce Cu uptake into roots, is associated with lignin synthesis, and further contributes to the increased tolerance to Cu in *Arabidopsis*^10^.

*LACs* are involved in regulating a series of metabolic pathways in plant development and growth; but the functional redundancy among members of the *LAC* family is pervasive^12,14^, which may result in functional divergence among the *LAC* family members in plants. However, such divergence has not yet been clearly described. Here, we investigated the *LAC* family in *P. euphratica* (desert poplar). This species grows mainly in western China and adjacent Middle-Eastern regions^24^ and exhibits strong tolerance to severe drought and salinity^25^. *P. euphratica* provides multiple ecosystem services as a natural barrier to the expansion of deserts, such as resisting sandstorms, regulating oasis climates, and even maintaining the ecosystem balance^24^. Thus, *P. euphratica* is widely considered a model woody plant for the study of the abiotic resistance mechanisms of trees^26^. *P. euphratica* has evolved many strategies to adapt to the severe desert environment^25^. Among them, the polymorphisms in its leaves^27^ and its hard wood are considered two important adaptive traits^28^ that may provide desert poplars with increased flexibility to adapt to desert environments^29^. For example, *P. euphratica* wood can accumulate substantial amounts of cellulose and lignin in its xylem. Within the xylem, the secondary cell wall (SCW) not only provides mechanical support for plants^30^ and acts as a defense barrier to pathogen and insect attacks^31,32^ but also provides a channel for the long-distance transportation of nutrients and water. Altogether with the polymorphisms in leaves, these traits allow the plants to exhibit physiological responses that help them adapt to extremely arid environments, including decreased photosynthetic activity, stomatal aperture control, and altered cell wall elasticity^33^. However, the molecular adaptive mechanisms underlying these physiological responses remain unknown.

In this work, we identified members of the *LAC* family in the *P. euphratica* genome and performed phylogenetic and evolutionary analyses. The *LAC2* gene, which has been subjected to positive selection, was chosen and used for genetic and functional analyses in *P. alba* var. *pyramidalis* and *Arabidopsis thaliana* (Col-0), respectively. Our study provides insights into the adaptive evolution of key genes that provide important genetic resources for the adaptation of desert poplar to extremely arid environments.

## Results

### Identification of *LAC* genes from the *P. euphratica* genome

**Fig. 1:**
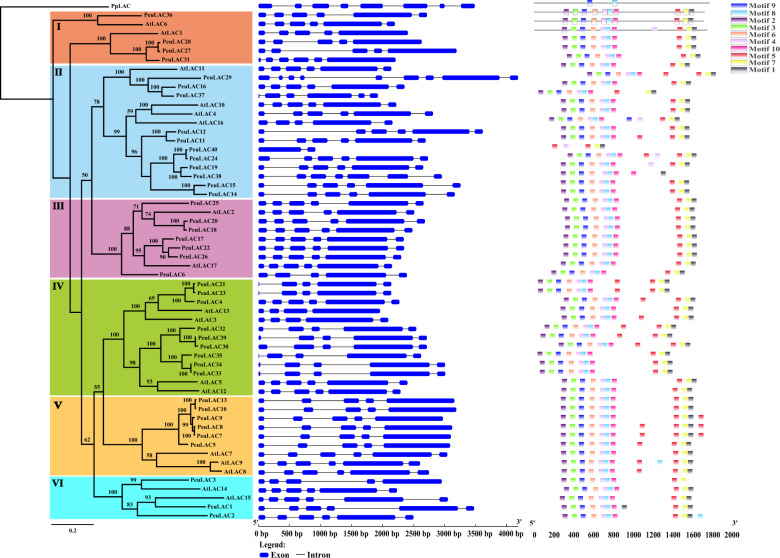
Phylogenetic tree, exon-intron structures, and conserved motifs of the *LAC* genes from *P. euphratica* (Peu) and *A. thaliana* (At). A maximum likelihood (ML) phylogenetic tree was generated with RaxML software using amino acid sequences; the *PpLAC* gene was used as an outgroup from *Physcomitrella patens*. All *LAC*s in the tree clustered into six clades (I–VI), which are shown with differently colored backgrounds. The lengths of the introns and exons are shown proportionally for each *LAC* gene. Exons are shown as solid blue boxes, and introns are shown as black lines. The motifs were determined using MEME and are shown as differently colored boxes. The numbers and colors represent motifs 1–10. The lengths of the motifs are indicated by the corresponding box size

We identified the *LAC* family through a BLASTp search using 17 *Arabidopsis* LAC protein sequences to BLAST against the *P. euphratica* genome. The Pfam and SMART databases were used to search for conserved domains, and 40 *LAC* genes with Cu-oxidase domains, named *PeuLAC1-PeuLAC40* (Table S1 and Supplementary Data 1), were identified for phylogenetic analyses. An ML tree was generated using RaxML, with *PpLAC* from *Physcomitrella patens* as the outgroup (Fig. 1). In the ML tree, all *LACs* clustered into six clades (I–VI). In each clade, *LACs* from *P. euphratica* had more copies than those from *Arabidopsis* and were grouped into 13 gene pairs with high bootstrap values (Fig. 1).

The gene structures of *PeuLACs* were investigated using GSDS (Fig. 1). The *PeuLACs* showed varying intron-exon patterns, and the exon number ranged from 2 to 9. A total of 10 conserved motifs in *PeuLACs* were detected using MEME (Fig. 1). The analyses of the biochemical characteristics showed that the molecular masses of PeuLACs ranged from 29.38 kDa (PeuLAC40) to 71.44 kDa (PeuLAC29), the predicted theoretical isoelectric points (pIs) ranged from 6.41 (PeuLAC2) to 9.82 (PeuLAC6), and most PeuLACs consisted of between 550 and 580 amino acid residues (Table S1). Signal peptides at the N-terminus were predicted in 25 PeuLACs. Furthermore, we predicted the phosphorylation sites and variable *N*- or *O*-glycosylation sites in all PeuLAC proteins (except PeuLAC40), indicating the potential for posttranslational modification (Table S1).

The tissue-specific expression patterns of the *PeuLAC* gene family in *P. euphratica* were investigated from RNA-seq data^34^. The results showed that *PeuLAC*s were expressed in various tissues (roots, leaves, xylem, and phloem) and were regulated by NaCl treatments (150 and 300 mM NaCl, respectively). However, in clade I (except *PeuLAC36*) and clade V, four gene pairs, *PeuLAC24/40*, *PeuLAC22/26*, *PeuLAC30/39*, and *PeuLAC33/34*, had similar expression patterns, while *PeuLAC16/37*, *PeuLAC19/38*, and *PeuLAC1/2* showed significant differences (Fig. S1). These results suggest not only possible functional redundancy between the gene pairs but also functional divergence among the duplicates.

### Analysis of the adaptive evolution of the *LAC* family in *P. euphratica*

**Fig. 2:**
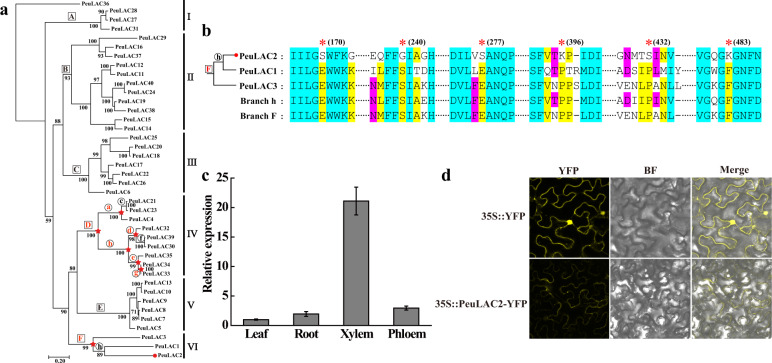
Adaptive evolution analysis of the LAC family in *P. euphratica*, expression pattern and subcellular localization of PeuLAC2. **a** A maximum likelihood (ML) phylogenetic tree of all 40 *PeuLACs*. All *PeuLAC* genes clustered into six clades (I–VI). Red letters and red stars represent the signals of positive selection detected in ancestral branches (branches D and F and subbranches a, b, d, e, and g). In branches A, B, C, and E and subbranches c, f, and h, no signals of positive selection were detected. The red circle represents the signal of positive selection detected in terminal branch *PeuLAC2*. **b** Sequence alignments and six positive selection sites. Alignments of *PeuLACs* in clade VI terminal branches (*PeuLAC1*, *PeuLAC2*, *PeuLAC3*) and ancestral branches (branch F and branch h) were generated by DNAMAN software. Positive selection sites are indicated with asterisks. **c** Expression of *PeuLAC2* examined by qRT-PCR in the leaves, roots, xylem and phloem of *P. euphratica*. **d** Subcellular localization of the PeuLAC2 protein; 48 h after transient expression in *Nicotiana benthamiana*, confocal images of PeuLAC2 fused to an N-terminal YFP and of the control 35S::YFP were obtained. YFP-only images, brightfield images, and merged images are shown

To examine whether some *PeuLAC*s experienced adaptive evolution, putative positive selection was investigated using a two-step branch-site model from CODEML in PAML^35^. Based on the ML tree (Fig. 2a), signals of positive selection were detected in branches D (clade IV, *P* < 0.001) and F (clade VI, *P* < 0.001) (Fig. 2a and Table 1). Further analysis using a BEB procedure revealed four sites (alignment positions 22, 65, 85, and 92) with posterior probabilities ≥ 0.99 that apparently underwent positive selection in the ancestral branch of branch F (clade VI) (Table 1). Then, we detected all terminal and ancestral branches in clade IV and clade VI, which exhibited positive selection in their ancestral nodes. In clade VI, we found a positive selection signal only in the terminal branch of *PeuLAC2* (*P* = 0.013, *ω*_2_ = 88.382) and at six sites (alignment positions 170, 240, 277, 396, 432, and 483) with posterior probabilities ≥ 0.80 (Fig. 2b and Table 1). In clade IV, positive selection was also detected in the ancestral branch of other branches (branches a, b, d, e, and g) (Fig. 2a and Table 1), but no positive selection signals were detected in the terminal branches of clade IV.

**Table 1:** Analysis of positive selection in *PeuLAC* family genes in *P. euphratica* using a branch-site PAML model

Branch-site Model	-ln *L*	2△ (ln *L*)	*P-*value	Parameter estimates	Positively selected sites
Dataset I: all *PeuLACs* (40 sequences)					
Branch F					
Null	6929.287611			*ω*_0_ = 0.11, *ω*_1_ = 1.0, *ω*_2_ = 1.0	
Alternative	6922.843499	12.89	<0.001	*ω*_0_ = 0.11, *ω*_1_ = 1.0, *ω*_2_ = 999.000	22Y (0.990*); 65S (0.999**); 85S (0.993**); 92D (0.993**)
Branch D					
Null	6931.431556			*ω*_0_ = 0.11, *ω*_1_ = 1.0, *ω*_2_ = 1.0	
Alternative	6920.259594	22.34	<0.001	*ω*_0_ = 0.11, *ω*_1_ = 1.0, *ω*_2_ = 999.000	
Dataset II: clade VI (3 sequences)					
Branch (terminal branch of *PeuLAC2*)					
Null	4739.621196			*ω*_0_ = 0.06, *ω*_1_ = 1.0, *ω*_2_ = 1.0	
Alternative	4736.546196	6.15	=0.013	*ω*_0_ = 0.07, *ω*_1_ = 1.0, *ω*_2_ = 88.382	170S (0.867*); 240G (0.856*); 277S (0.824*); 396K (0.842*); 432S (0.832*); 483K (0.890*)
Dataset III: clade IV (9 sequences)					
Branch a					
Null	5541.400987			*ω*_0_ = 0.07, *ω*_1_ = 1.0, *ω*_2_ = 1.0	
Alternative	5534.424466	13.95	<0.001	*ω*_0_ = 0.07, *ω*_1_ = 1.0, *ω*_2_ = 13.415	
Branch b					
Null	5537.882645			*ω*_0_ = 0.06, *ω*_1_ = 1.0, *ω*_2_ = 1.0	
Alternative	5532.622033	10.52	<0.001	*ω*_0_ = 0.07, *ω*_1_ = 1.0, *ω*_2_ = 4.560	
Branch e					
Null	5555.958312			*ω*_0_ = 0.07, *ω*_1_ = 1.0, *ω*_2_ = 1.0	
Alternative	5549.757667	12.40	<0.001	*ω*_0_ = 0.08, *ω*_1_ = 1.0, *ω*_2_ = 998.990	
Branch d					
Null	5556.551602			*ω*_0_ = 0.08, *ω*_1_ = 1.0, *ω*_2_ = 1.0	
Alternative	5549.891034	13.32	<0.001	*ω*_0_ = 0.08, *ω*_1_ = 1.0, *ω*_2_ = 998.997	
Branch g					
Null	5557.376628			*ω*_0_ = 0.08, *ω*_1_ = 1.0, *ω*_2_ = 1.0	
Alternative	5553.172610	8.41	<0.001	*ω*_0_ = 0.08, *ω*_1_ = 1.0, *ω*_2_ = 119.419	

Note: Codons identified by PAML as under positive selection along with Bayesian (BEB) analysis posterior probability for sites with *P* > 0.80 under branch-site models (*, 0.99 > *P* > 0.80; **, *P* > 0.99)

### *PeuLAC2* is expressed ectopically in *Arabidopsis* and poplar

To investigate the putative function of *PeuLAC2*, full-length *PeuLAC2* cDNA was cloned into the pBIB-BASTA-35S-GWR-GFP vector by the Gateway method. The resulting construct vector was then transformed into *Arabidopsis* and *P. alba* and used to generate 18 *PeuLAC2*-OE *Arabidopsis* lines and 16 *PeuLAC2*-OE *P. alba* lines, respectively. Under normal growth conditions, there were no significant changes in the phenotypes of transgenic poplar and *Arabidopsis* plants compared with those of WT plants. The expression level of *PeuLAC2* in the transgenic lines was analyzed by quantitative reverse transcription PCR (qRT-PCR) (Fig. S5). Of these transgenic lines, the At-OE4, At-OE6, and At-OE9 lines of *Arabidopsis* and the Pal-OE2, Pal-OE5, and Pal-OE8 lines of *P. alba*, which exhibited high, moderate, and low expression levels, respectively (Fig. S5), were selected for subsequent analyses.

The expression of *PeuLAC2* among tissues in *P. euphratica* was confirmed by qRT-PCR to occur mainly in the xylem (Fig. 2c). The subcellular localization of PeuLAC2 was examined in *N. benthamiana* epidermal cells. The fusion vector *35S::PeuLAC2-YFP* and the control *35S::YFP* were transiently expressed. Observation by confocal laser-scanning microscopy revealed that the yellow fluorescence of the 35S::PeuLAC2-YFP fusion protein was localized exclusively in the membrane of *N. benthamiana* epidermal cells, whereas the control YFP protein was localized in both the membrane and the nucleus (Fig. 2d).

### Drought tolerance in *Arabidopsis* and poplar with *PeuLAC2*-OE

Previous studies have shown that *LACs* participate in the plant response to abiotic stresses^12^. To investigate the role of *PeuLAC2* in drought tolerance, we investigated the *PeuLAC2* expression levels in the leaves, roots, xylem, and phloem of *P. euphratica* subjected to a drought treatment for 0 or 15 days. The results showed that the drought treatment significantly induced the expression of *PeuLAC2* in sapling leaves and xylem, while no significant change was observed in the roots and phloem (Fig. S2). Furthermore, we also assessed the drought sensitivity of transgenic plants. *PeuLAC2*-OE *Arabidopsis* plants were stressed by withholding water for 18 days and then rehydrated for 2 days. The survival rate was determined according to the standard that surviving plants could grow new leaves and grow normally. After 18 days without being watered, *PeuLAC2*-OE and WT *Arabidopsis* plants showed symptoms of drought-induced damage such as leaf rolling and wilting, but *PeuLAC2*-OE plants showed only slight symptoms and At-WT showed visible symptoms (Fig. S3a). After rewatering for 2 days, the At-OE4, At-OE6 and At-OE9 plants survived at rates of 63.9%, 62.5%, and 58.3%, respectively, which were all higher than the survival rate of At-WT (22.2%) (Fig. S3b). In terms of the water loss rate, the three At-OE plants maintained a reduced rate of water loss and showed higher drought tolerance than At-WT (Fig. S3c). Similar results were also found in terms of the leaf RWC (Fig. S3d). These results indicated that *PeuLAC2* overexpression improved drought tolerance in *Arabidopsis*.

To investigate whether *PeuLAC2* is involved in drought-induced oxidative stress, we examined the related parameters in *PeuLAC2*-OE and WT *Arabidopsis* plants treated by withholding water for 0, 12, and 15 days. The results showed that the MDA concentration was significantly higher in At-WT than in At-OE plants after 12 days without watering (Fig. S3e). The PRO level was lower in At-WT than in At-OE plants after being treated for 12 and 15 days (Fig. S3f). The H_2_O_2_ content was higher in At-WT than in At-OE plants after 15 days without watering, but this difference was not significant (Fig. S3g). The activities of POD and SOD, which contribute to avoiding oxidative damage induced by drought stress, increased significantly in the At-OE plants (Fig. S3h, S3i). These results indicated that *PeuLAC2* overexpression increased antioxidant enzyme activities and decreased oxidative damage under drought stress.

**Fig. 3:**
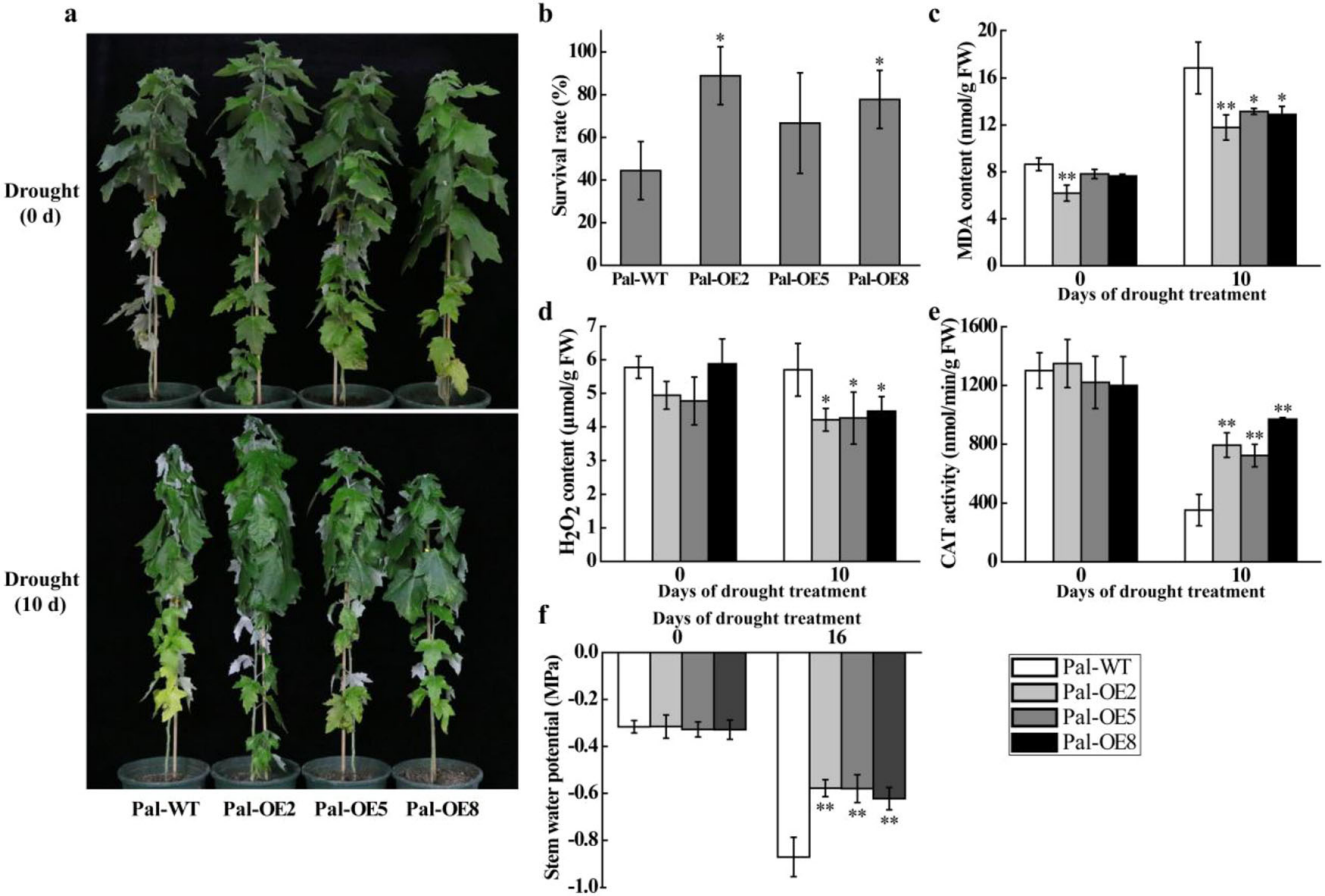
*PeuLAC2* overexpression improves drought tolerance in poplar. **a** Phenotypic comparison of Pal-WT and Pal-OE plants grown in soil under drought conditions (withholding water) for 0 days (as a control) and 10 days. **b** Statistical analysis of the survival rate in poplar after withholding water for 16 days followed by rewatering for 5 days. **c** to **d** Contents of MDA (**c**) and H_2_O_2_ (**d**) in Pal-WT and three Pal-OE plants were measured after 0 and 10 days of drought stress. **e** The activity of CAT in Pal-WT and three Pal-OE plants measured after 0 or 10 days of drought stress. **f** The stem water potential in Pal-WT and three Pal-OE plants measured after withholding water for 16 days or providing adequate water for 16 days. Three independent experiments were performed. Statistical analysis was performed with Student’s *t*-test (*P < 0.05, **P < 0.01); data are provided as means ± SDs. FW, fresh weight

We then analyzed drought tolerance in poplar that exhibited *PeuLAC2* overexpression. When the Pal-OE and Pal-WT plants were stressed for 10 days without watering, the Pal-WT plants showed severe symptoms of drought-induced damage, such as leaf rolling and wilting, but Pal-OE poplars wilted to a much lesser extent than Pal-WT poplars (Fig. 3a). After 16 days without watering followed by rewatering for 5 days, the survival rate of the Pal-OE plants was significantly higher than that of the Pal-WT plants (Fig. 3b). We also investigated drought-induced oxidative stress in poplars. When the Pal-OE and Pal-WT plants were treated for 10 days without watering, the Pal-OE plants displayed significantly lower MDA and H_2_O_2_ concentrations than the Pal-WT plants (Fig. 3c, d). The activity of CAT, which is the most important hydrogen peroxide-scavenging enzyme, was significantly higher in Pal-OE plants than in Pal-WT plants after 10 days without watering (Fig. 3e). These results indicated that *PeuLAC2* overexpression improved plant drought tolerance by reducing oxidative damage under drought stress.

**Fig. 4:**
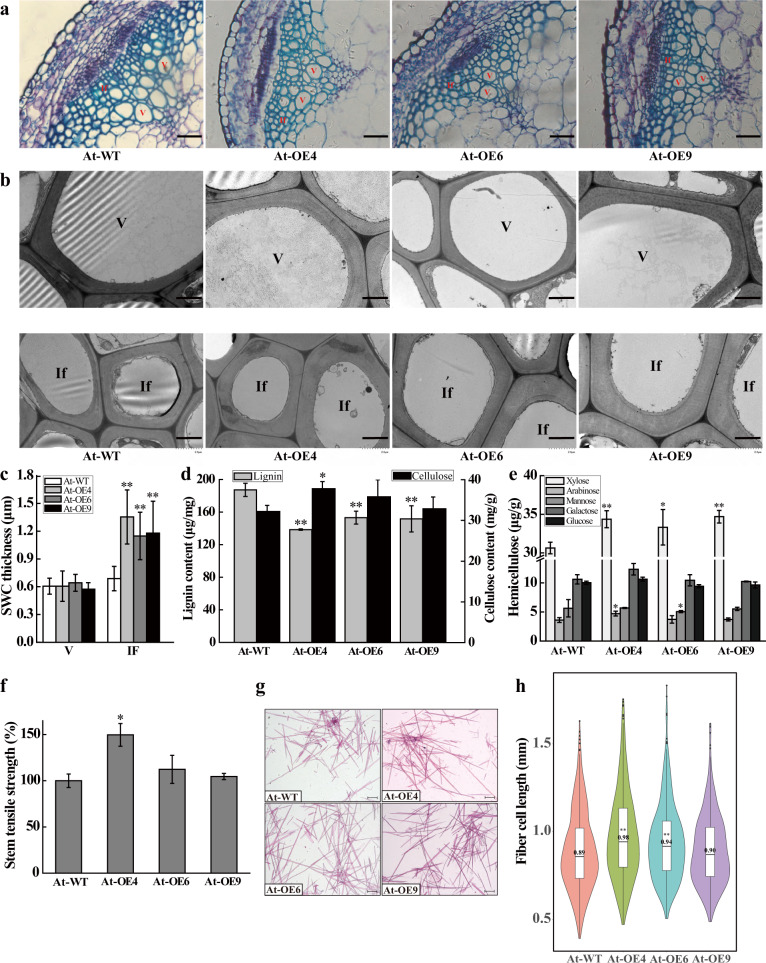
*PeuLAC2* overexpression enhances SCW thickness in fibers and affects SCW components in the inflorescence stem of *Arabidopsis*. **a** Cross-sections of *Arabidopsis* inflorescence stems in At-WT and three At-OE plants stained with 0.1% toluidine blue. V, vessel; If, interfascicular fiber cell. Bar = 20 μm. **b** Transmission electron micrographs (TEM) of the cell cross-sections shown in **a**. V, vessel; If, interfascicular fiber cell. Bar = 2 μm. **c** The SCW thickness was measured in the vessel cells and interfascicular fiber cells in **b** by ImageJ software, and at least 30 cells in each of three plants were measured. **d** to **e** SCW components analyzed in the *Arabidopsis* inflorescence stem. Contents of lignin, cellulose (**d**) and hemicellulose (**e**). **f** Tensile strength analysis of the *Arabidopsis* inflorescence stem. More than 20 plants in each line were measured. **g** Fiber cells in *Arabidopsis* inflorescence stems were disaggregated and stained with safranin T. Bar = 1 mm. **h** Statistical analysis of the fiber cell length in **g**; at least 500 fiber cells were measured in each plant genotype. Three independent experiments were performed. Statistical analysis was performed with Student’s *t*-test (*P < 0.05, **P < 0.01); data are provided as means ± SDs

**Fig. 5:**
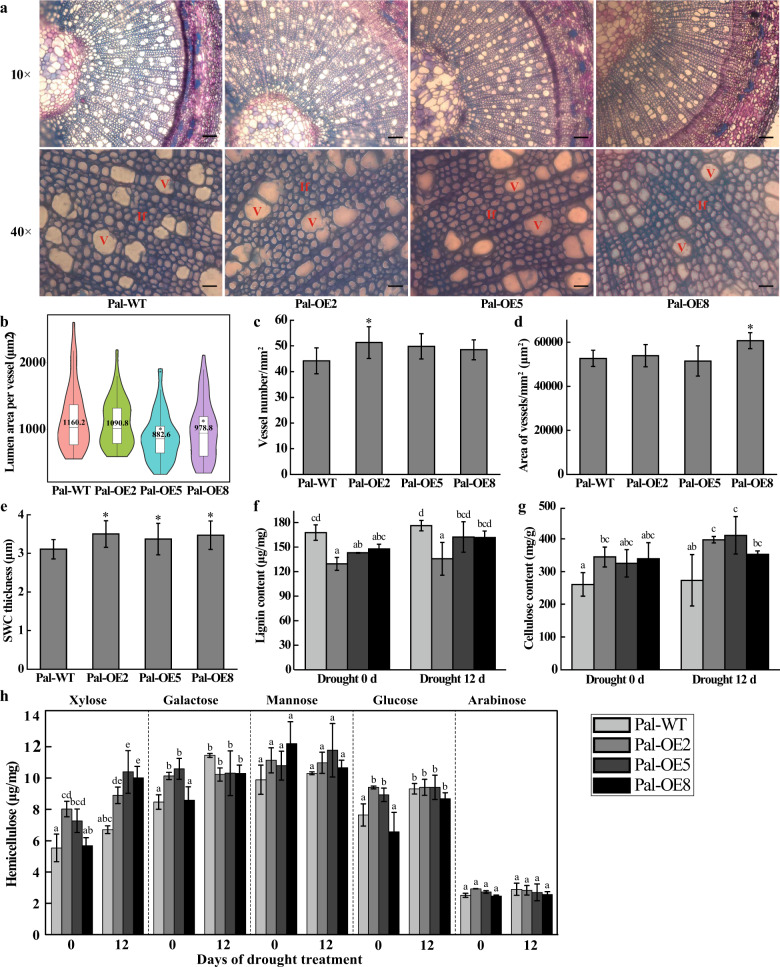
*PeuLAC2* overexpression enhances SCW thickness in fibers and affects the number and size of vessels in the xylem of *P. alba*. **a** Cross-sections of the fifth internode of the stem in Pal-WT and three Pal-OE *P. alba* plants stained with 0.1% toluidine blue and imaged at ×10 magnification, Bar = 100 μm and ×40 magnification, Bar = 20 μm. **b**–**d** Mean lumen area of individual vessels (μm^2^) (**b**), number of vessels per cross-sectional area (mm^2^) (**c**), and area of vessels (μm^2^) per cross-sectional area (mm^2^) (**d**) for the vessel cells from (**a**). More than 100 vessel cells in each of the three plants were measured. **e** SCW thickness of the xylem cells in **a**; at least 30 cells in each of three plants were measured. **f** to **h** SCW components in the *P. alba* stem analyzed under normal and drought conditions. Contents of lignin (**f**), cellulose (**g**), and hemicellulose (**h**). Three independent experiments were performed. Statistical analysis was performed with Student’s *t*-test (*P < 0.05, **P < 0.01); data are provided as means ± SDs

Previous studies have shown that higher stem xylem water potential can prevent hydraulic failure caused by drought and enhance drought tolerance^36^. We thus determined the effect of *PeuLAC2* overexpression on physiological changes in poplar, which may contribute to drought survival. The stem xylem water potential of *PeuLAC2*-OE poplars was higher than that of Pal-WT poplars under drought stress (Fig. 3f). We examined the morphology of poplar stem xylem cells in Pal-OE and Pal-WT plants. In *P. alba*, the stem xylem vessels in the Pal-OE plants were smaller than those in the Pal-WT plants (Fig. 5a, b), while the Pal-OE plants had a much higher vessel number per unit of area than the Pal-WT plants, especially Pal-OE2 (Fig. 5a, c). The area of vessels in the transverse section of the woody stem was higher in the Pal-OE plants than in the Pal-WT plants (Fig. 5d). However, in *Arabidopsis*, the stem xylem vessels, the vessel number per unit of area, and the area of vessels in the transverse section in the At-OE plants were not significantly different from those in the At-WT plants (Figs. 4a and Fig. S4). These responses may contribute to improving the water transport capacity of the plants.

### Determination of SCW thickness and composition of xylem cells and stem tensile strength in *Arabidopsis*

Previous studies have revealed that *PbLAC1* overexpression in *Arabidopsis* significantly increases the cell wall thickness in the xylem fibers and interfascicular fibers^15^. In this study, we generated paraffin sections to observe the thickness of the cell wall in both the interfascicular and xylem fibers of *Arabidopsis*. Toluidine blue staining in *Arabidopsis* revealed that the SCW thickening of the fiber cells in At-WT plants was weaker than that in At-OE (Fig. 4a). These results were further verified by TEM in *Arabidopsis* (Fig. 4b, c). The vessel cell thickness did not differ significantly between At-OE and At-WT plants (Fig. 4b, c).

*PeuLAC2* overexpression increased the SCW thickness in *Arabidopsis* regardless of whether it altered the cell wall composition. The examination of the cell wall composition revealed that in the At-OE4, At-OE6, and At-OE9 *Arabidopsis* plants, the lignin contents were 138.47, 153.19, and 151.71 μg/mg, respectively, which were significantly lower than those in At-WT (187.23 μg/mg) (Fig. 4d); the cellulose contents of the SCWs of these transgenic lines were 37.79, 35.80, and 32.87 mg/g, respectively, which were significantly higher than that in At-WT (32.19 mg/g) (Fig. 4d). Moreover, the content of xylose, an important component of hemicellulose, increased significantly in transgenic plants (34.36, 33.32, and 34.69 μg/g for At-OE4, At-OE6, and At-OE9, respectively) compared with that in At-WT plants (30.64 μg/g) (Fig. 4e), while the contents of other hemicelluloses, such as arabinose, mannose, galactose, and glucose, showed no significant differences (Fig. 4e).

The overexpression of *PeuLAC2* altered the cell wall composition and increased the cellulose and xylose contents of the interfascicular fibers and xylem cells in *Arabidopsis*. These fibers and cells are the main stem tissues supporting the upright growth of inflorescences. The tensile strength of the *Arabidopsis* inflorescence stem was measured, and the results indicated that the mechanical strength was higher in At-OE plants than in At-WT plants. The stem tensile strength of At-WT was defined as 100%, and in the three transgenic lines (At-OE4, At-OE6, and At-OE9), the stem tensile strengths were calculated as 149.72%, 112.35%, and 104.61%, respectively (Fig. 4f). Furthermore, the inflorescence stem structure was examined, and the results showed that the fiber cells in the transgenic plants were longer (average lengths of 0.98, 0.94, and 0.90 mm for At-OE4, At-OE6, and At-OE9, respectively) than those in the At-WT plants (0.89 mm) (Fig. 4g, h).

### Determination of SCW thickness and composition of xylem cells in poplar

*PeuLAC2* overexpression increased the SCW thickness and altered the cell wall composition of xylem cells in *Arabidopsis*; similar results were observed in poplar. We determined the SCW thickness and cell wall composition of xylem cells in the Pal-OE and Pal-WT poplars. The results indicated that the thickening of the SCWs of the fiber cells in the Pal-OE poplars was significantly greater than that in the Pal-WT poplars (Fig. 5a, e). *PeuLAC2* overexpression also reduced the vessel area and increased the vessel number per unit of area and the area of the vessels in the transverse section in poplar plants (Fig. 5b–d). The determination of the cell wall composition showed that *PeuLAC2* overexpression also decreased the lignin content and increased the cellulose content in *P. alba* (Fig. 5f, g). However, with regard to hemicellulose, the xylose content increased in Pal-OE compared with that in Pal-WT, while the glucose, arabinose, mannose, and galactose contents in the Pal-OE plants showed no significant differences from those in the Pal-WT plants (Fig. 5h). These results showed that *PeuLAC2* was involved in regulating SCW biosynthesis in interfascicular fiber and xylem cells.

We also determined the composition of the SCWs in poplar plants under drought conditions. For the drought treatment, two-month-old Pal-OE and Pal-WT poplar saplings were subjected to drought for 0 and 12 days, respectively, and the SCW composition of those sapling stems was examined. The results showed that *PeuLAC2* overexpression significantly decreased the lignin content and increased the cellulose content of the SCWs in *P. alba* under drought for 0 and 12 days, respectively (Fig. 5f, g). However, for hemicellulose, *PeuLAC2* overexpression significantly increased the xylose contents of SCWs under drought treatment (Fig. 5h); the contents of galactose, mannose, glucose, and arabinose in both Pal-OE and Pal-WT plants increased under drought conditions, but the differences in the increases between genotypes were not significant (*P* > 0.05) (Fig. 5h). These results further indicated that *PeuLAC2-*OE affects the contents of lignin, cellulose, and hemicellulose in SCWs, which may affect SCW functioning and might be involved in drought tolerance.

### RNA-seq analysis of *PeuLAC2*-OE plants to evaluate drought tolerance

**Fig. 6:**
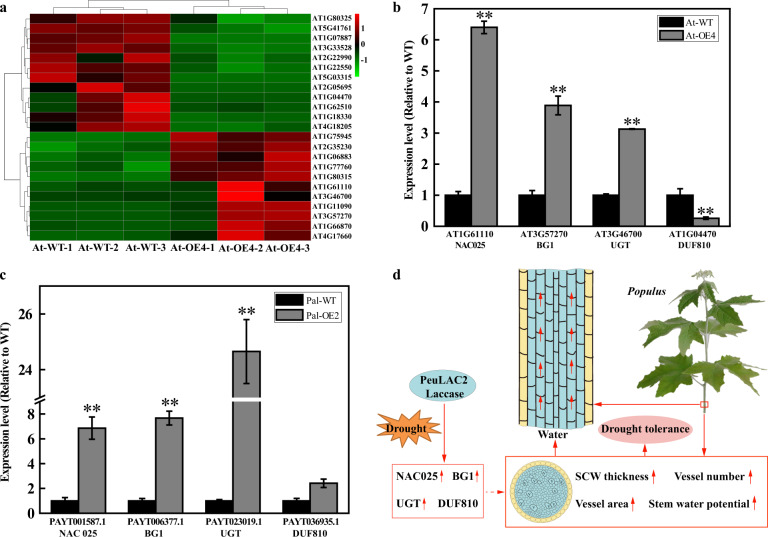
*PeuLAC2* overexpression regulates the expression of genes related to drought tolerance and SCW formation in *Arabidopsis*. **a** Cluster heat map showing the differentially expressed genes (DEGs) in At-OE plants (adjusted *P*-value < 0.1); red indicates high relative expression, and green indicates low relative expression. **b** qRT-PCR analysis of the effect of *PeuLAC2* on the expression of drought tolerance- and SCW-related genes (*NAC025*, *BG1*, *UGT*, and *DUF810*) in *Arabidopsis* under drought treatment. **c** qRT-PCR analysis of the orthologous genes in poplar (**b**) under drought treatment for 10 days. Three independent experiments were performed. Statistical analysis was performed with Student’s *t*-test (*P < 0.05, **P < 0.01); data are provided as means ± SDs. **d** A proposed model of the regulation of drought tolerance and SCW thickening in Populus by *PeuLAC2*. *PeuLAC2* overexpression changed the transcription program for drought tolerance and SCW formation by upregulating the expression of *NAC025*, *BG1*, and *UGT* and downregulating the expression of *DUF810* to enhance drought tolerance and SCW thickening. Arrows: regulation directly confirmed by this experiment. Dashed arrow: regulation suggested by this experiment. Red arrows indicate promotion

To further reveal the potential regulatory network through which *PeuLAC2* is involved in drought tolerance, we conducted an RNA-seq analysis of *PeuLAC2*-OE *Arabidopsis* plants. After drought stress for 12 days, only 15 DEGs (adjusted *P*-value < 0.05) were identified, with seven upregulated genes and eight downregulated genes (Table S3). In the drought-treated At-OE plants, a total of 23 DEGs were detected (adjusted *P*-value < 0.1), with 11 upregulated genes and 12 downregulated genes (Fig. 6a and Table S3). Among the upregulated genes, *NAC025* (AT1G61110), *UGT* (AT3G46700), and *BG1* (AT3G57270) are associated with stress resistance and the synthesis of the cell wall components cellulose and xylose, which influence the cell wall structure. Among the downregulated genes, *AT1G04470*, a member of the DUF810 family, is related to stomatal responses as well as salt and drought tolerance (Table S3). qRT-PCR analysis further confirmed the expression levels of these genes in *PeuLAC2*-OE *Arabidopsis* plants. The results indicated that the expression of *NAC025*, *BG1*, and *UGT* was activated and that the expression of *DUF810* (AT1G04470) was suppressed in transgenic *Arabidopsis* lines (Fig. 6b). We were interested in determining whether these up- and downregulated genes exhibited similar expression patterns in poplar plants under drought stress. Based on the *NAC025*, *BG1*, *UGT*, and *DUF810* genes in *Arabidopsis*, we identified the corresponding orthologous genes in poplar: *PAYT001587.1* (*NAC025*), *PAYT006377.1* (*BG1*), *PAYT023019.1* (*UGT*), and *PAYT036935.1* (*DUF810*). qRT-PCR results revealed that the expression of *PAYT001587.1* (*NAC025*), *PAYT006377.1* (*BG1*), and *PAYT023019.1* (*UGT*) was activated but that the expression of *PAYT036935.1* (*DUF810*) did not change significantly in poplar plants under drought conditions (Fig. 6c).

## Discussion

Drought can seriously limit the growth and development of plants. The laccase protein family is considered to be important in plant responses to various stresses^10,12,20,21,23^. Previous evidence, including spatial-temporal expression patterns and expression regulation models, has shown that the functional redundancy among members of the *LAC* family is pervasive in *Arabidopsis*^12,14^. The functional redundancy of duplicated genes is widespread and provides an important source of genetic novelties^37,38^ upon which natural selection may act to contribute to the maintenance of partial gene redundancy^38^. Increasing amounts of experimental data indicate that redundant pairs are often associated with proteins involved in signaling and posttranslational protein modifications, such as phosphorylation and ubiquitination^39^. However, less experimental evidence that could elucidate the roles of natural selection in redundant gene evolution is available.

### Natural selection may contribute to the retention of *LAC* gene redundancy

*LACs* in *P. euphratica* have experienced multiple gene duplications, most of which likely occurred specifically in *P. euphratica*. Forty *LACs* from *P. euphratica* that clustered into six clades were identified based on amino acid sequence alignment with LAC-like multicopper oxidases, and most clades exhibited wider expansion than was observed in *Arabidopsis LACs*. *LACs* in *P. euphratica*, e.g., the gene pairs *PeuLAC24* and *PeuLAC40*, *PeuLAC19*, and *PeuLAC15* in clade II and the gene pairs *PeuLAC30* and *PeuLAC39*, *PeuLAC33*, and *PeuLAC34* in clade IV, had diverse sequences but exhibited highly conserved expression patterns after salt treatment (Fig. S1), showing apparent functional redundancy across a wide variety of tissues. Functional redundancies were also found among clades; for example, *AtLAC4*, *AtLAC11*, and *AtLAC17* in clade III and *AtLAC15* in clade VI have been shown to be related to lignin synthesis^11,13,14^. Evolutionary analysis showed that positive selection signals were detected in clade IV (*P* < 0.001) and clade VI (*P* < 0.001) (Fig. 2a and Table 1). In particular, in clade IV, both gene pairs exhibited redundant functions, indicating that natural selection might be involved in the retention of gene redundancy. In clade VI, three *PeuLACs* experienced different selective pressures and were expressed in different patterns. *PeuLAC2* was subjected to positive selection, and six amino acid residues experienced adaptive evolution. The orthologous gene *AtLAC14* in *Arabidopsis* is considered to be associated with stress responses^12^, while *AtLAC15* is related to lignin synthesis and cell wall structure^13^; these results indicate that *LACs* in clade VI have diversified significantly in function. In addition, among all PeuLAC proteins (except PeuLAC40), phosphorylation sites and variable *N*- or *O*-glycosylation sites have been found, indicating potential posttranslational modifications^17^. It is obvious that the expanded *LACs* in *P. euphratica* have experienced functional redundancy that would be associated with posttranslational protein phosphorylation modifications^39^.

### *PeuLAC2* improves drought tolerance by enhancing water transport capacity

As a multigene family, *LACs* have been verified to be associated with lignin polymerization^11,14,17^ and with responses to various stresses in plants^10,12,20,21,23^. Here, we found that *PeuLAC2*-OE lines had reduced water loss rates during drought stress (Fig. S3c). Consistent with the water loss rate, the leaf RWC was higher in the *PeuLAC2*-OE plants than in the At-WT lines after drought treatment (Fig. S3d). These results indicated that *PeuLAC2* overexpression reduced water loss by increasing RWC in *Arabidopsis*. In addition, the activities of POD and CAT were both enhanced in transgenic lines (Fig. S3f and Fig. 3e), resulting in reduced H_2_O_2_ accumulation (Fig. 3d) and indicating an enhanced antioxidant defense system in transgenic plants. The accumulation of high levels of reactive oxygen species (ROS) induced by drought stress not only damages cell membranes but also results in cell death^40^. The overexpression of *PeuLAC2* enhances antioxidant defense system activity to effectively protect membrane integrity and avoid damage to plants.

*LACs* are related to the formation of cell wall structures and lignin metabolism^10,11,15,16^. In this study, the *PeuLAC2-*OE plants showed significantly increased SCW thickness in xylem cells in *PeuLAC2*-OE *P. alba* (Fig. 5e) and *Arabidopsis* (Fig. 4b, c). This result suggests that *PeuLAC2* participates in the development of the SCW in xylem cells. In terms of the SCW components, the cellulose content was significantly higher in *PeuLAC2-*OE plants than in WT plants (Figs. 4d and 5g). Similar results were also found for another SCW component, hemicellulose. For example, xylose is one of the most abundant components of hemicellulose, and the xylose content was significantly increased in *PeuLAC2*-OE *Arabidopsis* plants (Fig. 4e). The contents of other hemicellulose components, including arabinose, mannose, galactose, and glucose, were not significantly different between genotypes (Fig. 4e). These results revealed that *PeuLAC2* enhanced the cellulose and xylose contents and further increased SCW thickness in *Arabidopsis*. Both cellulose and xylose participate in cell wall formation. Celluloses, as the main component of the cell wall, have great mechanical strength and lead to high stem tensile strength in plants by increasing the length of fiber cells in the inflorescence stem. In addition, the hemicellulose and cellulose in plant cell walls are primarily consumed by the fungi that cause brown rot decay^41^; thus, the overexpression of *PeuLAC2* might significantly improve plant resistance to fungi.

Furthermore, in plant conducting cells, the size and structure of stem xylem vessels can affect the water transport capacity of plants^42^. Hydraulic conductivity in xylem is associated with the water potential and vessel diameter^36,42^. Under drought conditions, plants can regulate their hydraulic conductance as an adaptation to enhance drought tolerance^4,43^. The upward water transport through the xylem fails when a decrease in water potential reduces hydraulic conductivity; this phenomenon is called xylem cavitation or embolism^36^. Vessels with smaller diameters can endure lower water potentials to prevent xylem cavitation^42^. In *PeuLAC2*-OE *P. alba* plants under drought stress, the stem xylem water potential was higher (Fig. 3f), the vessel lumen area was smaller, and the number of vessel cells and area of vessels were much greater (Fig. 5a–d) than those in the Pal-WT controls; these differences contributed to enhancing the water transport capacity and led to improved drought tolerance in the transgenic poplars.

Increasing evidence indicates that laccase enzymes exhibit dual functions to catalyze both lignin biosynthesis and degradation^44^. These processes can be affected by multiple limiting factors, such as the pH^45^, three-dimensional structure^46^, and C-terminus features^46,47^, which can all be changed by *PeuLAC2* overexpression in plants. For example, among all of the LACs from *P. euphratica*, PeuLAC2 had the lowest theoretical isoelectric point (6.41) (Table S1), an additional motif 8 at the C-terminus (Fig. 1), and six adaptive amino acid sites (Fig. 2b and Table 1). These features together might contribute to altering its function to negatively regulate lignin content, improve the water transport capacity and further enhance drought tolerance in plants.

### *PeuLAC2* induces the expression of many genes involved in water transport in conducting cells

A comparative transcriptome analysis of At-OE plants under drought conditions provided insight into the molecular mechanisms involved in the *PeuLAC2*-mediated drought-stress response and changes in SCW thickness. We identified DEGs from At-OE lines, and qRT-PCR analysis further confirmed the differential expression of *AT1G04470*, *NAC025*, *β-1,3-GLUCANASE*1 (*BG1*), and *UGT* (Fig. 6b). *AT1G04470*, a member of the DUF810 family, was suppressed in the At-OE plants under drought stress (Fig. 6b and Table S3). In *Arabidopsis*, the DUF810 family gene and the *PATROL1* mutation (*patrol1*) impaired the stomatal opening response^48^. The rice gene *OsDUF810.7* is involved in tolerance to salt and drought stresses^49^. UDP-glycosyltransferases (*UGTs*) glycosylate a wide range of metabolites and phytohormones in response to biotic and abiotic stresses. *UGT74E2* overexpression increases the tolerance of *Arabidopsis* to drought and salinity stresses and reduces water loss in plants, while 11 *UGTs* are upregulated in response to hydrogen peroxide stress in plants deficient in catalase^50^. *UGTs* and *β-1,3-GLUCANASE1* are also involved in cellulose and xylose synthesis, which influences the SCW structure^51^. NAC proteins play diverse roles in plant growth, development, and defense^52^, e.g., *NAC*, *VND6*, *VND7*, *NST1*, and *SND1* are involved in SCW synthesis^53^. Based on the four genes in *Arabidopsis*, we identified the corresponding orthologous genes in poplar. The expression of the four genes in transgenic *Arabidopsis* and poplar was verified using qRT-PCR (Fig. 6b, c). *NAC025*, *BG1*, and *UGT* were upregulated in both *Arabidopsis* and poplar plants, showing similar expression patterns. However, *DUF810* was downregulated in *Arabidopsis*, but there was no significant change in its expression in poplar. One possible explanation for this difference is that *DUF810* shows different expression patterns in herbaceous and woody plants under drought stress. Therefore, we propose a simple model of how *PeuLAC2* regulates drought tolerance by improving the water transport capacity in poplar (Fig. 6d). In the model, *PeuLAC2* overexpression changes the transcription of several genes associated with SCW structure formation and stress responses, such as *NAC025*, *BG1*, *UGT*, and *DUF810*, to enhance drought tolerance.

Taken together, from an evolutionary perspective, redundancies due to gene duplication likely increased the ability of desert poplar (*P. euphratica*) to evolve multiple adaptive traits. For example, *P. euphratica* wood is rigid and dense, and substantial amounts of lignin and cellulose accumulate in the xylem; together with the SCW, this accumulation not only provides mechanical support for plants^30^ and a defense barrier^31,41^ but also prevents excessive water evaporation from the plant, thus increasing the likelihood of the plant’s survival in desert ecosystems^25,32^. Collectively, our results provide evidence that *PeuLAC2*, underwent adaptive evolution, contributes to the thickening of SCWs by enhancing their cellulose and xylose contents, which may enhance the water transport capacity of *P. euphratica* and improve its tolerance to drought stress. Our data also contribute to clarifying the genetic mechanisms underlying drought tolerance in *P. euphratica* and suggest that *PeuLAC2* could be an attractive candidate gene for molecular breeding programs for trees.

## Methods and materials

### Identification of *LACs* in *P. euphratica*

*P. euphratica* genome databases were used to collect amino acid sequences of *LAC* (*PeuLAC*) genes. A BLASTp search was performed in the whole-genome database using all 17 amino acid sequences of *Arabidopsis* LACs as queries^12^. SMART software (http://smart.embl-heidelberg.de/) and the Pfam database (http://pfam.xfam.org) were used to validate potential LACs identified in the *P. euphratica* genome. Protein queries that did not contain the known conserved domains were removed. A maximum likelihood (ML) phylogenetic tree was generated using amino acid sequences with RaxML software. Through the comparison of each putative *PeuLAC* coding sequence with its genomic sequence by the Gene Structure Display Server (GSDS v2.0; http://gsds.cbi.pku.edu.cn/), we identified the *PeuLAC* gene structures. The possible conserved motifs of the *PeuLAC* genes were predicted using MEME server v4.11.4 with the default parameters (http://meme-suite.org/tools/meme), except for the maximum number of identified motifs, which was set as 10.

The physical and chemical characteristics of the PeuLACs were analyzed using ProtParamtool (http://web.expasy.org/protparam). Potential glycosylation sites were predicted using the YinOYang 1.2 server (http://www.cbs.dtu.dk/services/YinOYang/) and the NetNGlyc 1.0 server (http://www.cbs.dtu.dk/services/NetNGlyc/). Putative signal peptide cleavage sites were analyzed using the SignalP 5.0 server (http://www.cbs.dtu.dk/services/SignalP/). Phosphorylation sites were analyzed using the NetPhos 3.1 server (http://www.cbs.dtu.dk/services/NetPhos/).

### Molecular evolutionary analysis

Codon-based ML models were used to estimate the rates of nonsynonymous substitutions (*d*_N_) and synonymous substitutions (*d*_S_) and the *d*_N_/*d*_S_ ratio (omega, *ω*) in the CODEML program in PAML 4.7^35^. The ratio of nonsynonymous to synonymous substitutions (*ω*) was used to estimate the change in selective pressure, where *ω* = 1, *ω* < 1, and *ω* > 1 correspond to neutral, purifying, and positive selection, respectively. The *PeuLAC* ML tree was used as an input tree in all analyses (Fig. 2a). Branch-site model A was used to estimate positive selection, in which the model *ω* could vary among sites along specific lineages. Positive selection analysis for every gene in each foreground lineage was performed using the modified branch-site model A. The likelihood ratio test (LRT) statistic (2∆*L*) approximating a chi-square distribution was used to compare nested likelihood models. Bayes empirical Bayes (BEB) analysis^35^ was used to identify all positively selected sites in the branch-site models with posterior probabilities ≥0.80.

### Plant materials

*Arabidopsis* seeds of WT (Col-0, At-WT) and three transgenic lines of *PeuLAC2*-overexpression (At-OE) were surface-sterilized using 70% (v/v) ethanol for 45 s and then with 1% sodium hypochlorite for 10 min; finally, they were washed five times in sterilized water. The surface-sterilized seeds were vernalized at 4 °C for 2 days in the dark to break dormancy and were grown on MS medium containing 1% (w/v) sucrose and 0.8% (w/v) agar (pH 5.8) in a growth chamber at 22 °C and 60% relative humidity (RH) under a 16/8 h (light/dark) cycle with 4500 lux light. For poplars, *PeuLAC2* transgenic overexpression lines (Pal-OE) and WT (Pal-WT) *P. alba* saplings were propagated by in vitro microcutting. For the clonal propagation of sterilized transgenic and WT *P. alba* saplings, shoot segments of 2–3 cm were cut and cultivated on MS medium in a growth chamber at 23–25 °C and 60% relative humidity (RH) under a 14/10 h (light/dark) cycle under 4500 lux light.

### Plasmid construction and plant transformation

Total RNA was extracted from *P. euphratica* leaves and used for reverse transcription analysis. To generate the OE plants, the full-length 1704-bp coding sequence of *PeuLAC2* was amplified with specific primers (Table S2) and then cloned into the donor vector pDONR/Zeo via the Gateway method (Invitrogen, Germany). The obtained entry clones were used to transfer the target sequence into the destination vector. In this study, the binary destination vector *pBIB-BASTA-35S-GFP* with the *CaMV 35S* promoter and BASTA herbicide resistance was used to express *35S::PeuLAC2* in plants. *Agrobacterium tumefaciens* strain GV3101 with the construct *35S::PeuLAC2* was used to transform WT *Arabidopsis* by floral dip transformation^54^. The transformed plants were identified by T2 progeny that were resistant to BASTA, and T3 transgenic plants were confirmed using PCR. For poplar, *Agrobacterium*-mediated infiltration of leaf discs was used to generate stably transgenic *P. alba* plants^55,56^. Positive transgenic poplar plants were identified using BASTA herbicide, and PCR genotyping with specific primers for the *PeuLAC2* gene (Table S2) was performed to identify the positive transgenic plants.

### Subcellular localization

The coding sequence of *PeuLAC2* was cloned into the vector *35S::pEarleyGate-YFP* via Gateway technology (Invitrogen, Germany) with subcellular primers (Table S2). The construct *35S::PeuLAC2-YFP* and empty vector *35S::YFP* were introduced into *A. tumefaciens* strain GV3101. The constructs were grown in Luria-Bertani (LB) medium with 20 μM acetosyringone (AS). When the optical density was 600 nm (OD_600_) = 0.5, the cultures were resuspended in injection buffer (liquid MS medium with 10 mM MgCl_2_, 150 μM acetosyringone, and 10 mM MES, pH 5.8). Then, resuspended cultures of *35S::PeuLAC2-YFP* and *35S::YFP* were injected into 3-week-old *Nicotiana benthamiana* leaves. A confocal laser-scanning microscope (TCSSP8, Leica) was used to observe YFP fluorescence at 488 nm emission 48 h after injection. Tissues infected with *35S::YFP* were used as negative controls.

### Analysis of drought-stress tolerance

For the drought treatment, the At-OE and At-WT *Arabidopsis* seedlings were cultured on MS medium. After 5 days, the seedlings were transferred into pots containing equal amounts of soil by weight for 2 weeks with adequate watering. Drought treatment was performed by withholding water for 12–18 days, and well-hydrated plants were used as controls. Watering resumed when the At-WT plants showed a fatal dehydration phenotype. After rewatering for 2 days, the survival rate of the stressed plants was calculated. Three independent experiments were performed. Each independent experiment contained six pots of At-WT and At-OE plants, respectively, and each pot contained nine *Arabidopsis* seedlings. We then examined water loss and the leaf relative water content (RWC). The rosette leaves of 4-week-old *Arabidopsis* plants were detached and placed into a glass culture dish at 22 °C and 30–40% RH. Then, the weight of the detached rosette leaves was measured every 1 h for 8 h. Water loss was calculated as the percentage of the leaf fresh weight that was lost. The RWC was assessed using rosette leaves from 2-week-old plants after withholding irrigation for 12 days, and well-hydrated plants were used as controls. Three biological replicates were performed.

We also evaluated the drought resistance of transgenic *P. alba* plants. Five-week-old poplar saplings were transferred into the soil in pots containing equal amounts of soil by weight and grown for 5 months with sufficient watering. Saplings at the same growth stage were subjected to drought stress implemented by withholding water, and well-hydrated plants served as controls. After 10 days, signs of leaf wilting and necrosis were observed. After 16 days, the plants were rewatered and maintained under well-watered conditions for 5 days, and their survival rate was calculated. To examine the stem water potential, poplar plants were cultured under well-hydrated and unwatered conditions for 16 days. According to the manufacturer’s instructions, the stem water potential was measured with a SAPS II Water Potential System (SEC). Three independent drought resistance experiments were performed. Each independent experiment included nine Pal-WT and Pal-OE saplings, respectively.

The proline (PRO), malondialdehyde (MDA), and hydrogen peroxide (H_2_O_2_) contents and antioxidant capacities, including the catalase (CAT), superoxide dismutase (SOD) and peroxidase (POD) activities, were measured by spectrophotometry as described in the protocols of the kits from Suzhou Comin Biotechnology (www.cominbio.com). Three biological replicates and three technical replicates were performed.

### Determination of SCW thickness and chemical components

For the determination of SCW thickness, *Arabidopsis* inflorescence stems were cut into 2–3 mm segments from 0.5 cm above the rosette leaves and then fixed for cell wall observation under a transmission electron microscope (TEM; HITACHI H-7650) according to the manufacturer’s protocols^57^. ImageJ (https://imagej.nih.gov/ij/) was used to measure the SCW thickness, and at least 30 cells in each of three plants were measured.

The chemical components of the SCW, such as its lignin, cellulose, and noncellulosic polysaccharide contents, were determined using previously described methods^58–60^. *Arabidopsis* inflorescence stems and 2-month-old poplar stem segments between the 2nd and 5th internodes were harvested, ground into a fine powder in liquid nitrogen, and used to generate alcohol insoluble residue (AIR) for the determination of the lignin, cellulose, and noncellulosic polysaccharide contents. Three biological replicates and three technical replicates were performed.

### Cross-sectioning and histological staining

*Arabidopsis* inflorescence stems and the fifth internode of stems from 5-month-old *P. alba* plants were cut into 0.5–1 cm segments, fixed in FAA solution, and embedded in paraffin. A rotary microtome (RM2235, Leica) was used to section the embedded fragments to a thickness of 12 μm. The sections were stained with 0.1% toluidine blue and observed using an optical microscope (Zeiss, Germany). ImageJ software was used to analyze the images to quantify the morphological parameters of the xylem cells, and at least 30 xylem cells in each of three plants were measured.

### RNA-seq analysis

For RNA-seq, water was withheld from 2-week-old *PeuLAC2*-OE and At-WT *Arabidopsis* plants for 15 days. The rosette leaves of five independent plants under drought stress and well-watered conditions were collected. Total RNA for RNA-seq was extracted by BIOMARKER (Beijing, China). To trim the reads, we removed adapter sequences, low-quality sequences, low-complexity sequences, and reads with many (> 5%) unknown bases. The TAIR10 *Arabidopsis* genome was used to align the resultant clean reads with the HISAT2 program (v2.0.4). The gene expression level and fragments per kilobase of transcripts per million fragment mapped (FPKM) values were quantified and calculated using RSEM (v1.2.12). Analysis of the differentially expressed genes (DEGs) was performed using the DESeq2 R package. Genes with an adjusted *P*-value < 0.05 and fold change (FC) ≥ 2 (|log2 (FC)| ≥ 1) were considered differentially expressed by DESeq2.

Quantitative RT-PCR was performed to examine gene expression levels using TB Green^®^ *Premix Ex Taq™* (TaKaRa) with a real-time PCR detection system (MX3005P, Agilent). Gene expression levels were normalized using ACTIN2 as an internal control. The relative expression levels of the target genes were calculated according to the 2^−△△CT^ method^61^. All experiments were performed with five biological replicates and three technical replicates.

### Statistical analyses

Statistical analyses to test the data from all experiments for significant differences were performed using SPSS version 16.0. Data are expressed as the means ± SDs based on at least three independent biological replicates. Differences were considered significant at *P* ≤ 0.05 according to Student’s *t*-test.

## Supplementary information

Supplementary Information
