# Supplementary material for: A gene that underwent adaptive evolution, LAC2 (LACCASE), in Populus euphratica improves drought tolerance by improving water transport capacity
Source: Hortic Res. 2021 Apr 1;8:88. doi: 10.1038/s41438-021-00518-x (PMC8016922; doi:10.1038/s41438-021-00518-x)
Supplement: Supplementary file 1 — Supplementary Information [file 41438_2021_518_MOESM1_ESM.docx]

**A gene that underwent adaptive evolution, *LAC2* (LACCASE), in *Populus euphratica* improves drought tolerance by improving water transport capacity**

Zhimin Niu^1^, Guiting Li^1^, Hongyin Hu^1^, Jiaojiao Lv^1^, Qiwei Zheng^2^, Jianquan Liu^1^, Dongshi Wan^1^

^1^State Key Laboratory of Grassland Agro-Ecosystem, School of Life Sciences, Lanzhou University, Lanzhou, Gansu, P. R. China

^2^Laboratory of Cell Biology, College of Life Science and Technology, Huazhong Agricultural University, Wuhan, P. R. China

Corresponding author:

Prof. Dongshi Wan

State Key Laboratory of Grassland Agro-Ecosystem,

School of Life Sciences, Lanzhou University, Lanzhou, 730000, Gansu, P. R. China.

Fax: +86-931-8914288;

Tel. +86-931-8914305.

E-mail: wandsh@lzu.edu.cn.

**Running title**：The adaptive evolution of *LAC2* from *P. euphratica* improved drought tolerance

**Highlights:**

1. A *LAC2* gene in *P. euphratica* underwent positive selection;

2. *PeuLAC2*-OE thickens the secondary cell wall;

3. *PeuLAC2*-OE improves drought tolerance in plants by enhancing their water transport capacity.

**Supplementary information：**


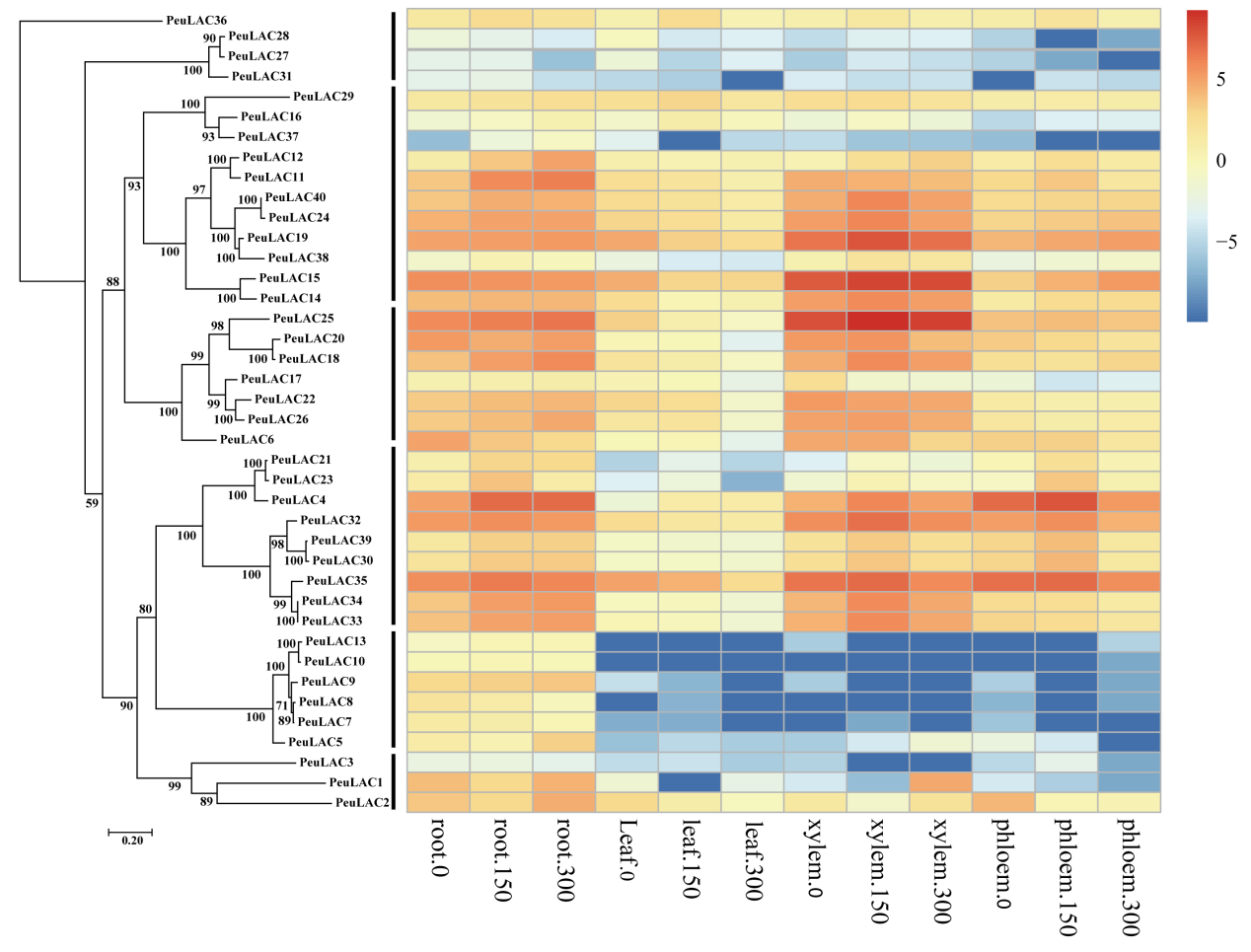


**Fig. S1. Cluster heat map showing the expression patterns of *PeuLAC* gene family.** The tissue-specific expression patterns of the *PeuLAC* gene family in *P. euphratica* were examined from RNA-Seq data under normal growth conditions and NaCl treatments (150 and 300 mM NaCl, respectively).

**
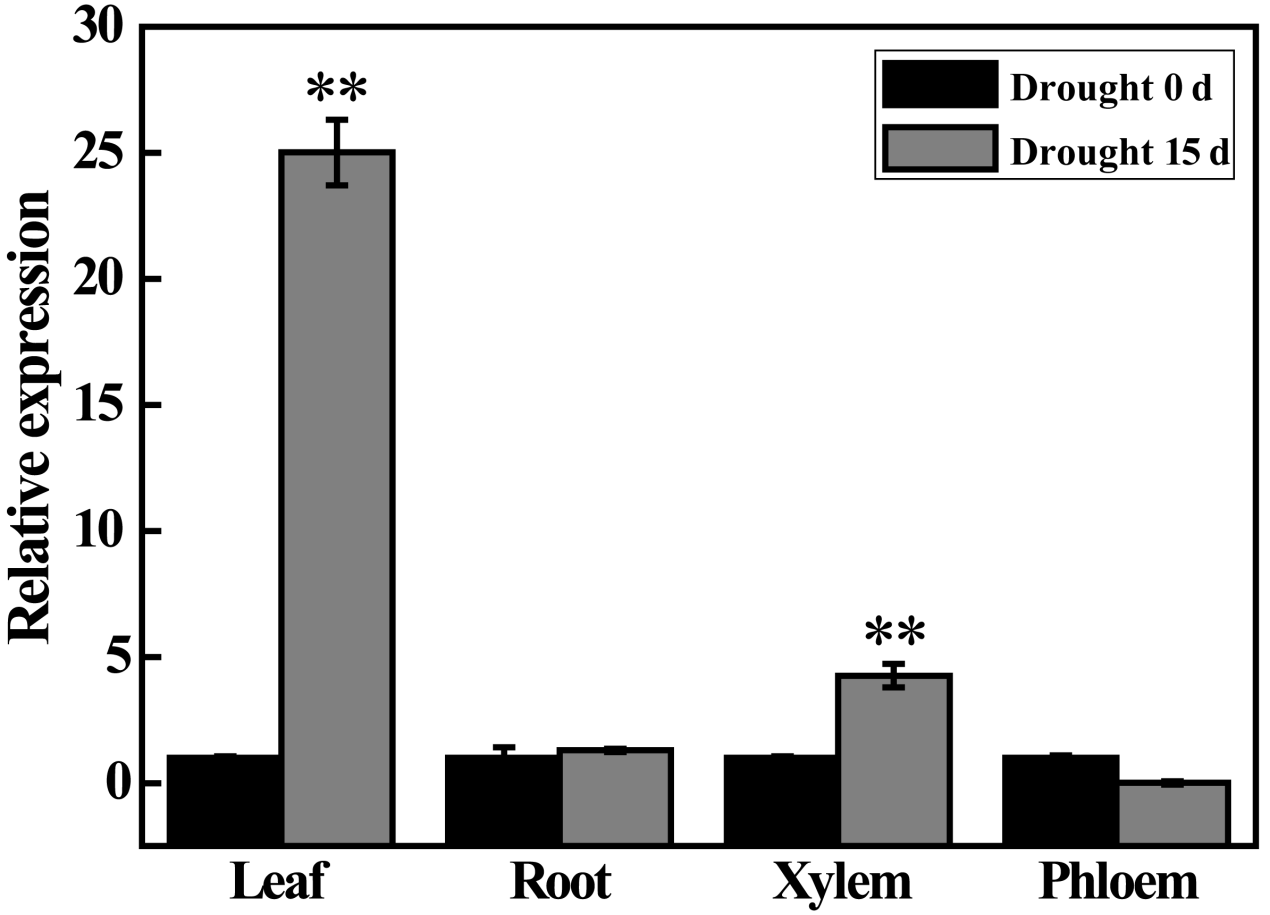
**

**Fig. S2. The expression patterns of *PeuLAC2* were examined using qRT-PCR in leaves, root, xylem, and phloem of *P. euphratica* under drought treatment for 0 and 15 days, respectively.** Three independent experiments were performed. Statistical analysis was performed with Student’s t-test (*P < 0.05, **P < 0.01); data are provided as means ± SDs.


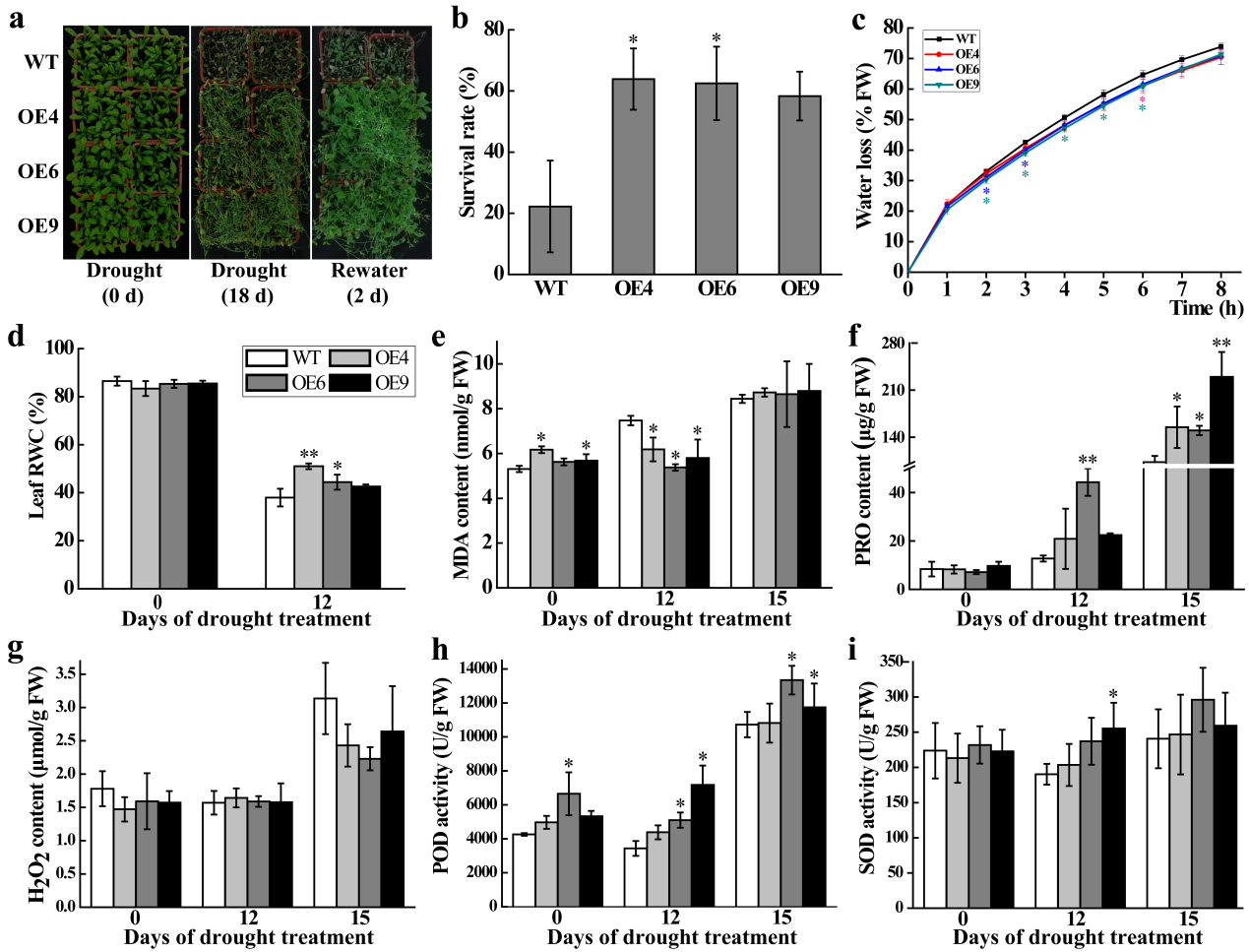
**Fig. S3. *PeuLAC2* overexpression improved drought tolerance in *Arabidopsis*. (a)** Phenotypic comparison of the At-WT and At-OE plants grown in soil withholding water for 0, 18 days and rewatering for 2 days. **(b)** Statistical analysis of survival rate after 2 days of rewatering. **(c)** Water loss assay of rosette leaves in the At-WT and At-OE plants. **(d)** RWC of the At-WT and At-OE plants under normal growth conditions and after water was withheld for 12 days. **(e)**-**(g)** Content of MDA (e), PRO (f) and H_2_O_2_ (g) in the At-WT and three At-OE plants were measured after 0, 12 or 15 days of drought stress, respectively. **(h)**-**(i)** Antioxidant enzyme activity of POD (h) and SOD (i) in the At-WT and three At-OE plants was measured after 0, 12 or 15 days of drought stress, respectively. Three independent experiments were performed. Statistical analysis was performed with student’s t-test (*P < 0.05, **P < 0.01); data are provided as means ± SDs.


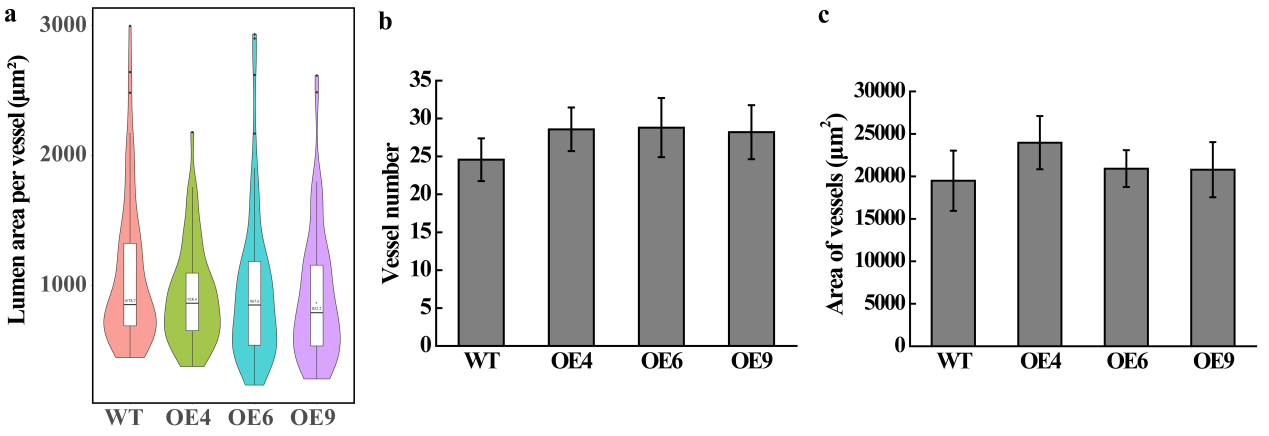


**Fig. S4. *PeuLAC2* overexpression affects the number and size of vessels in xylem of *Arabidopsis*.** **(a)** to **(c)** Mean lumen area of individual vessels (μm^2^) (a), number of vessels per cross-sectional area (mm^2^) (b), and area of vessels (μm^2^) per cross-sectional area (mm^2^) (c) using vessel cells from Fig. 4a were analyzed. More than 100 vessel cells in each of three plants were measured. Statistical analysis was performed with student’s t-test (*P < 0.05, **P < 0.01); data are provided as means ± SDs.


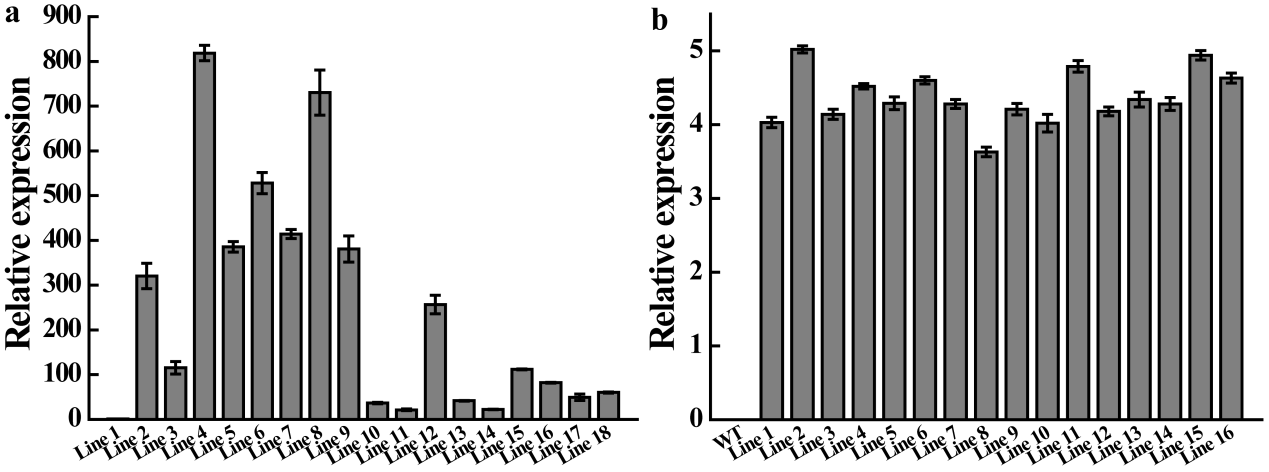


**Fig. S5. qRT-PCR determined the expression levels of *PeuLAC2* overexpression lines.** **(a)** The expression levels of *PeuLAC2* overexpression *Arabidopsis* lines. **(b)** The expression levels of *PeuLAC2* overexpression *P. alba* lines. Three independent experiments were performed. Data are provided as means ± SDs.

.

**Table S1. Physical, chemical characterization and the prediction of signal peptide of 40 PeuLACs**

| **Designate name** | **Sequence ID** | **Amino acid length (aa)** | **MW (kD)** | ***p*I** | **Signal peptide length (aa)** | **Cleavage site** | **Potential glycosylation sites** | | **Potential phosphorylation sites** | | |
| --- | --- | --- | --- | --- | --- | --- | --- | --- | --- | --- | --- |
|  |  |  |  |  |  |  | **N-glyc** | **O-glyc** | **Serine** | **Threonine** | **Tyrosine** |
| PeuLAC1 | CCG009016.1 | 567 | 63.46 | 7.28 | 26 | CQA-IV | 7 | 11 | 24 | 23 | 13 |
| PeuLAC2 | CCG012411.1 | 567 | 62.60 | 6.41 | 26 | CMA-QS | 9 | 19 | 23 | 15 | 10 |
| PeuLAC3 | CCG002095.1 | 525 | 58.83 | 9.34 | 24 | ANG-KI | 8 | 11 | 14 | 25 | 7 |
| PeuLAC4 | CCG000986.1 | 576 | 64.22 | 9.15 | 32 | SEA-ET | 8 | 10 | 18 | 18 | 7 |
| PeuLAC5 | CCG023995.1 | 562 | 61.45 | 6.56 | 22 | ASA-AI | 10 | 12 | 17 | 20 | 7 |
| PeuLAC6 | CCG011063.1 | 540 | 59.71 | 9.82 | - | - | 13 | 11 | 18 | 22 | 5 |
| PeuLAC7 | CCG003618.1 | 569 | 62.25 | 6.83 | 22 | ASA-AI | 10 | 15 | 20 | 17 | 6 |
| PeuLAC8 | CCG004956.1 | 569 | 62.19 | 7.02 | 22 | ASA-AI | 10 | 15 | 22 | 15 | 6 |
| PeuLAC9 | CCG016842.1 | 569 | 62.39 | 7.03 | 22 | ASA-AI | 9 | 13 | 17 | 19 | 5 |
| PeuLAC10 | CCG016726.1 | 569 | 62.15 | 8.05 | 22 | ASA-AI | 8 | 15 | 20 | 17 | 7 |
| PeuLAC11 | CCG030766.1 | 556 | 60.87 | 9.10 | 23 | VES-MV | 12 | 5 | 12 | 24 | 5 |
| PeuLAC12 | CCG022529.1 | 556 | 61.06 | 8.89 | 23 | VES-MV | 10 | 6 | 13 | 24 | 3 |
| PeuLAC13 | CCG022479.1 | 539 | 58.89 | 8.36 | - | - | 8 | 10 | 19 | 19 | 7 |
| PeuLAC14 | CCG000168.1 | 556 | 60.99 | 8.63 | 23 | VQC-KV | 9 | 13 | 13 | 26 | 5 |
| PeuLAC15 | CCG020485.1 | 555 | 60.91 | 8.72 | 22 | VEC-KV | 12 | 11 | 14 | 27 | 6 |
| PeuLAC16 | CCG017447.1 | 561 | 62.71 | 8.96 | 27 | AEA-AV | 12 | 9 | 11 | 17 | 4 |
| PeuLAC17 | CCG011922.1 | 581 | 64.42 | 9.45 | 31 | AVA-IT | 13 | 5 | 16 | 22 | 6 |
| PeuLAC18 | CCG030825.1 | 576 | 63.41 | 9.09 | 31 | VAG-AK | 15 | 9 | 19 | 23 | 7 |
| PeuLAC19 | CCG014170.1 | 557 | 60.84 | 9.34 | 22 | VEC-RI | 10 | 13 | 17 | 24 | 2 |
| PeuLAC20 | CCG017004.1 | 576 | 63.55 | 9.31 | 31 | VAG-AK | 17 | 7 | 17 | 24 | 5 |
| PeuLAC21 | CCG029512.1 | 489 | 53.84 | 6.72 | - | - | 7 | 10 | 17 | 12 | 5 |
| PeuLAC22 | CCG032374.1 | 581 | 64.04 | 9.07 | 31 | ALA-VS | 13 | 10 | 19 | 20 | 8 |
| PeuLAC23 | CCG009228.1 | 489 | 54.06 | 7.24 | - | - | 7 | 9 | 15 | 13 | 5 |
| PeuLAC24 | CCG023973.1 | 580 | 63.24 | 9.27 | - | - | 9 | 13 | 26 | 24 | 3 |
| PeuLAC25 | CCG011067.1 | 580 | 63.85 | 9.22 | 33 | AAA-IT | 17 | 8 | 15 | 25 | 8 |
| PeuLAC26 | CCG006502.1 | 581 | 64.05 | 9.27 | 31 | ALA-IT | 12 | 9 | 14 | 21 | 6 |
| PeuLAC27 | CCG008664.1 | 579 | 64.44 | 8.72 | 27 | CSS-QT | 2 | 12 | 21 | 22 | 7 |
| PeuLAC28 | CCG023637.1 | 579 | 64.38 | 8.21 | 27 | CSS-QT | 2 | 13 | 23 | 23 | 6 |
| PeuLAC29 | CCG022244.2 | 645 | 71.44 | 6.58 | - | - | 9 | 7 | 20 | 21 | 6 |
| PeuLAC30 | CCG025535.1 | 559 | 62.29 | 8.45 | - | - | 9 | 16 | 24 | 24 | 9 |
| PeuLAC31 | CCG012778.1 | 593 | 66.00 | 9.23 | - | - | 3 | 14 | 21 | 20 | 9 |
| PeuLAC32 | CCG000252.1 | 511 | 56.87 | 8.72 | 28 | MHG-IR | 7 | 11 | 20 | 19 | 11 |
| PeuLAC33 | CCG020400.1 | 499 | 54.95 | 7.20 | - | - | 11 | 14 | 23 | 17 | 8 |
| PeuLAC34 | CCG025534.1 | 499 | 54.95 | 7.20 | - | - | 11 | 14 | 23 | 17 | 8 |
| PeuLAC35 | CCG000253.1 | 491 | 53.91 | 6.49 | - | - | 10 | 13 | 21 | 20 | 8 |
| PeuLAC36 | CCG010816.1 | 572 | 63.49 | 7.24 | 24 | IHA-AP | 7 | 7 | 18 | 23 | 9 |
| PeuLAC37 | CCG033203.1 | 444 | 49.18 | 8.77 | - | - | 8 | 5 | 10 | 20 | 5 |
| PeuLAC38 | CCG014171.1 | 476 | 52.17 | 9.06 | - | - | 6 | 9 | 17 | 19 | 2 |
| PeuLAC39 | CCG020401.1 | 498 | 55.22 | 8.64 | - | - | 7 | 16 | 19 | 21 | 9 |
| PeuLAC40 | CCG023974.1 | 271 | 29.38 | 9.43 | - | - | 7 | 8 | 10 | 11 | - |

**Table S2.** **Primer sequences used in this study**

| Primers | Sequence (5'-3') |
| --- | --- |
| PeuLAC2_OE_F | AAAAAAGCAGGCTTCATGGGGATAAGCAGGTTG |
| PeuLAC2_OE_R | CAAGAAAGCTGGGTTCTAGGAGCAAGATGGCAG |
| PeuLAC2_qRT-PCR_F | CTCACAGTTGTTGGGCAAGATG |
| PeuLAC2_qRT-PCR_R | TGGGAGGACTGGGAGTGGAATAG |
| PeuLAC2_subcellular_F | ATGGGGATAAGCAGGTTGGGT |
| PeuLAC2_subcellular_R | GGAGCAAGATGGCAGGGACGC |
| NAC025_qRT-PCR_F | GGAAGTATCCAAATGGGGTTAGG |
| NAC025_qRT-PCR_R | GCAGTGCTAAGGTTACCATCAGTG |
| BG1_qRT-PCR_F | CCTCTACACTTACTTCAGCTACG |
| BG1_qRT-PCR_R | CAGTCTCCGCCACAACTATTTCC |
| UGT_qRT-PCR_F | TGTAGTCAACAAAAGAACAGCCTC |
| UGT_qRT-PCR_R | GGTTTCTGCTTGTTCAGCCATTC |
| AT1G04470_qRT-PCR_F | GTTACATTCCTACGCTTCCTCCAC |
| AT1G04470_qRT-PCR_R | GTGAGAGGGATTTGTTGAGAGAGTG |
| ACTIN2_F | GCACCCTGTTCTTCTTACCG |
| ACTIN2_R | AACCCTCGTAGATTGGCACA |
| PAYT001587.1_qRT-PCR_F | GGACAAGGAGGATTCAATGGAGG |
| PAYT001587.1_qRT-PCR_R | TACTGATGAGGAGGGAGGGTGCGTG |
| PAYT006377.1_qRT-PCR_F | GCCAATCTTGACACTGTTTACGC |
| PAYT006377.1_qRT-PCR_R | CCCCAATGTTTCTCAAGTTCAG |
| PAYT023019.1_qRT-PCR_F | CTTCGGGAGTCATTTGGAACACG |
| PAYT023019.1_qRT-PCR_R | GCAACACTCCCAAAACTCACAT |
| PAYT036935.1_qRT-PCR_F | CAATCTGTGCTTTGCTTGGGTGC |
| PAYT036935.1_qRT-PCR_R | GACGCTGATAATGCTAAAGGTAAG |
| CYC063_F | CCACGAACCGCAGAGAACTT |
| CYC063_R | CTCCATAGATTGATTCTCCTCCG |

**Table S3. Summary of transcriptome analysis of the OE plants compared with WT plants in *Arabidopsis* under drought stress**

| **Locus** | **Gene name** | **Gene model type** | **Involved in** | **Log2FoldChange** | **Up-Down** | **Adjusted P-value** | **Description** |
| --- | --- | --- | --- | --- | --- | --- | --- |
| AT1G07887 |  | antisense_long_noncoding_rna |  | -5.675 | Down | 1.96E-115 | Natural antisense transcript overlaps with AT1G53480 |
| AT1G80315 |  | protein_coding |  | 4.455 | Up | 8.47E-52 | Hypothetical protein |
| AT1G75945 |  | protein_coding |  | 5.938 | Up | 8.18E-50 | Hypothetical protein |
| AT2G05695 |  | long_noncoding_rna |  | -8.889 | Down | 4.590E-17 |  |
| AT2G35230 | HAIKU1, IKU1 | protein_coding | endosperm development, regulation of seed growth | 1.894362789 | Up | 1.950E-08 | Contains a plant-specific VQ motif. Involved in endosperm growth and seed size determination. IKU1 is expressed in the early endosperm and its progenitor, the central cell.IKU1 interacts with MINI3 in the yeast two-hybrid system. |
| AT3G33528 |  | protein_coding |  | -2.286 | Down | 4.310E-06 | Hypothetical protein |
| AT1G11090 | MAGL1 | protein_coding | acylglycerol lipase activity | 9.299 | Up | 6.620E-06 | alpha/beta-Hydrolases superfamily protein |
| AT4G18205 | PUP21, PURINE PERMEASE 21 | protein_coding | purine nucleoside transmembrane transporter activity | -3.272 | Down | 1.390E-05 | Nucleotide-sugar transporter family protein |
| AT1G22550 | NPF5.16 | protein_coding | low-affinity nitrate transmembrane transporter activity, oligopeptide transmembrane transporter activity, peptide transmembrane transporter activity, peptide:proton symporter activity | -1.303 | Down | 2.690E-05 | Tonoplast localized pH dependent, low affinity nitrogen transporter.In shoots, expressed in leaf veins and mesophyll. In roots, GUS activity was detected in root vascular stele. More highly expressed in roots |
| AT4G17660 |  | protein_coding | phosphorylation | 3.686 | Up | 7.100E-05 | Protein kinase superfamily protein |
| AT1G18330 | EARLY-PHYTOCHROME-RESPONSIVE1, EPR1, RVE7 | protein_coding | Circadian rhythm, regulation of transcription, DNA-templated, response to cadmium ion, response to ethylene, response to salt stress | -1.675 | Down | 0.000598 | EARLY-PHYTOCHROME-RESPONSIVE1 |
| AT5G41761 |  | protein_coding |  | -1.560 | Down | 0.001481 | Hypothetical protein |
| AT1G77760 | GNR1, NIA1, NITRATE REDUCTASE 1, NR1 | protein_coding | nitrate assimilation, nitric oxide biosynthetic process, oxidation-reduction process,response to herbicide, response to light stimulus | 1.369 | Up | 0.003105 | Encodes the cytosolic minor isoform of nitrate reductase (NR). Involved in the first step of nitrate assimilation, it contributes about 15% of the nitrate reductase activity in shoots. Similar to molybdopterin oxidoreductases at the N-terminus, and to FAD/NAD-binding cytochrome reductases at the C-terminus. Cofactors: FAD, heme iron (cytochrome B-557), and molybdenum-pterin. |
| AT1G66870 |  | protein_coding |  | 4.803 | Up | 0.007404 | Carbohydrate-binding X8 domain superfamily protein |
| AT1G04470 | DUF810 | protein_coding | domain of Unknown Function 810 (DUF810) family | -7.997 | Down | 0.019906 | Stomatal responses, salt and drought tolerance |
| AT5G03315 |  |  |  | -3.776 | Down | 0.055109 | Natural antisense transcript overlaps with AT5G21920 |
| AT1G61110 | ANAC025, NAC025, NAC25 | protein_coding | embryo development ending in seed dormancy, plant-type cell wall modification involved in multidimensional cell growth | 3.368 | Up | 0.067686 | Plant-type cell wall modification, NAC transcription regulator. Regulates endosperm cell expansion during germination. |
| AT3G57270 | β-1,3-GLUCANASE 1, BG1, | protein_coding | carbohydrate metabolic process, defense response | 3.180 | Up | 0.073385 | Encodes a member of glycosyl hydrolase family 17, Involved cellulose synthesis |
| AT1G62510 |  | protein_coding |  | -3.069 | Down | 0.074995 | Bifunctional inhibitor/lipid-transfer protein/seed storage 2S albumin superfamily protein |
| AT1G80325 |  | antisense_long_noncoding_rna |  | -1.487 | Down | 0.078372 | Natural antisense transcript overlaps with AT1G80320 |
| AT1G06883 |  | long_noncoding_rna |  | 5.009 | Up | 0.081772 |  |
| AT2G22990 | SCPL8, SINAPOYLGLUCOSE 1, SNG1 | protein_coding | phenylpropanoid metabolic process, proteolysis, secondary metabolic process | -1.303 | Down | 0.081772 | Sinapoylglucose:malate sinapoyltransferase. Catalyzes the formation of sinapoylmalate from sinapoylglucose. Mutants accumulate excess sinapoylglucose. |
| AT3G46700 |  | protein_coding | UDP-glycosyltransferase activity | 4.021 | Up | 0.087141 | UDP-Glycosyltransferase superfamily protein, involved in lignin synthesis |

**Supplementary data 1: Amino acid sequence information used for the construction of phylogenetic trees and sequence alignments of *PeuLAC2*.**

>PeuLAC1

MLPIMRVLAFQILRFLLFGGFLCCQAIVHHTFVVKDAPYTRLCSTKNIMTVNGQFPGPTLYVTKGETIIVDVINKSPHNITIHWHGVKQPKYPWSDGPEYITQCPIQPGGKFSQRVIFSDEEGTLWWHAHSDWTRATVYGAIVIYPKKGTEYPFPTPHADVPIILGEWWKKDIFEIFDQFRASGADPNVSDAYTINGQPGDLYPCSKSDTFKLSVDCGKTYLLRLINAALQDILFFSITDHQVTVVGTDASYTKPLKVDYVAISPGQTIDVLLEANQPLDHYYMAAKVYSSANGVQYDNTTTTAIVQYNGNYTPSSTPSLPYLPYFNDTTASVNFTGRLRSLADNNHPIHVPLSISTPLFFTVSVNRFTCANTSCGATQSRLAASVNNISFQTPTRMDILRAYYNQINGVYGDHFPDKPPLFFNFTADSIPLMIYETPSKGTEVKVLEYNSTVEIVFQGTNVAAGTDHPMHIHGTSFYVVGWGFGNFDKDKDPLRYNLVDPPLQNTIAVPKNGWSAIRFKATNPGVWFVHCHLERHLSWGMVMAFIIKNGKGKTARMLPPPPYMPPC

>PeuLAC2

MGISRLGFIVGLIWFMAMDWQVLCMAQSNVHHYNFVVQNAQFTRLCETKTMLTVNGSFPGPTIHARKGDTIFVNVHNEGDYGVTIHWHGVKQPRNPWSDGPENITQCPIQPGKNFTYEIILSDEEGTLWWHAHSDWSRATVHGAIVISPARGTTYPFPAPYAEQTIIIGSWFKGDVKAVIDEAVATGGGPNISNSLTINGQPGDLYPCSEEDTYRLKVNSGRTYLLRVINAVMNEEQFFGIAGHSLTVVGQDAAYIKPITTNYIMITPGQTMDILVSANQPPSYYYIASHSFSDGAGVAFDNTTTTAIFQYNGNYSRPSAIPLPVLPIFNDTAAAENYTSRVRGLASRDHPVNVPQTINRRLYVTVSLNFLPCTEATCTGSNRLAASMNNVSFVTKPIDILQAYYRSINGVFDADFPSEPQKYFNFTGNMTSINVATARGTKVTMLNYGEAVEIVLQGTNLLAEMNHPIHLHGFSFYLVGQGKGNFNNETDPKSYNLIDPPEINTVALPRSGWAAIRFVANNPGVWFIHCHLEKHSSWGMDTVLIVRNGRTRAQSMRPPPASLPSCS

>PeuLAC3

MVKILRLLGFIVSLIIQNYTTANGKIHHHKFVVKSASFTRLCTTKEILTVNGKFPGPTLEAYTGDELRVTVYNRAKYNITLHWHGARQVRNPWSDGPEYITQCPIQPGRRFNYKITLTTEEGTIWWHAHNSWARATVHGALIIYPKHGSHYPFPKPHAEFPIILGEWWKKDVMKIPGDANITGGEPTLSAAFTINGEPGYMYPCSKAGTFKMMVEQGKTYLLRIINAVLDENMFFSIAKHKLTIVGKDGCYLKPFTSDYLMITPGQTMDVLFEANQPPSHYSMASRAYSSAFGAGFDNTTTTAIVEYHGIFHLLQSPHVSPLPPYNGTQASTDFTKQFRSLVKANVPQKVNTRLFFTISVNLLNCSTNKPCAGPFGKRFAASINNISFVNPPSLDILQAYYYGVAGVFKRNFPRKPPNEFNYTVENLPANLLTPSFGTKVRVLEYNASVEIILQGTNILAADNHPIHLHGYNFYVVGKGFGNFDPNKDPSKYNLDDPPEETTVGVPHNGWAVIRFKADNPGKKLK

>PeuLAC4

METRNLTVKQVSYRLFLSIFVIFSFQAHFSEAETHYREFVIQAKPVKRLCRTHNTITVNGLFPGPTLEVRDGDSLVIKAVNNARYNVTLHWHGIRQLRNPWADGPDRITQCPIQPGRSYTYRFTIENQEGTLWWHAHSKWLRATVYGALIIHPKLGSPYPFPMPRTEIPILLGEWWDRNPMDVLRLADFTGAAPNISDAYTINGQPGDLYRCSKQETVRFPVGSGETILLRVINAALNQELFFGVANHKLTVVAVDAAYTKPFTTSVIMISPGQTTDVLLTADQTPGQYYMAAHAYNSANAPFDNTTTTAILEYKTAPCNAKKGKQSTPIFPRLPGFNDTNSAIAFTSGLRSPSKVKVPLQIDENLFFTVGLGLINCPNPNSPRCQGPNGTRFAASINNVSFVLPKRNSLMQAYYQGQPGIFTTDFPPVPPVKFDYTGNVSRGLWQPVKATKLYKLKFGAKVQIVFQDTSIVTVEDHPMHLHGYHFAVIGSGFGNFNPQTDPARFNLIDPPYRNTIGAPPGGWVAIRFEADNPGIWLLHCHLDSHLNWGLGMAFLVENGVGKLQSVQPPPLDLPRC

>PeuLAC5

MLRLLFWLTCALVFLASSVASAAIVEHSFHVKNLTVRRLCTEQVVTAVNGSLPGPTLRVHEGDTLKVHVFNKSPYNMTLHWHGVFQLLSAWADGPDMVTQCPIQPGGKYTYQFKILKQEGTLWWHAHVSWLRATVYGALIIRPRSGHPYPFPKPDKEVPILFGEWWNANVVDVENQALASGAAPNISDAFTINGLPGDLYPCSQNRIFTLKVQKGKTYLLRIINAALNNELFFKIANHNMTVVAVDAGYTVPYVTGVVVIGTGQTVDVLLAADQEVGSYYMAANAYASAAGTLFDNTTTRGIVVYEGAPSSATPIMPLMPAFNDTPTAHKFFTNITGLAGGPHWVPVPRQIDEHMFVTVGLGLSICPTCSNGTQVSASMNNFSFVSPTTLSMLQAFFFNVSGIYTPDFPDTPPIKFDYTNASINTLNPSLLITPKSTSVKVLKYNSTVEMVLQNTAILGVENHPMHLHGFNFHVLAQGFGNYDPVKDPKKFNLVNPQSLNTIGVPVGGWAVIRFIANNPGVWFMHCHLDVHLPWGLATAFVVENGPTEDSTLPPPPADLPQC

>PeuLAC6

MQNVTRMCRTKSIVTVNGQIPGPRIIAREGDRLLIKVVNHVQYNVTLHWHGIRQLRSGWADGPAYVTQCPIQTGQSYIYNFTVTGQRGTLFWHAHISWLRATLHGPIVILPKKGVSYPFPQPHKEVPIIFGEWWKADTEKIISQALQTGGAPNISDAYTINGHPGLLYNCSAKDTFKLKVKPGKTYLLRLINAALNDELFFSIANHSLTVVEADAVYVKPFKTHIVLITPGQTTNVLLMAKAKAPNSTFLMAARPYATGPASFDNTTTAGILEYDHNPSATNSKSKNKKLPLLKPSLPVFNDTTFATKFVKKIRSLANARFPAKVPKKVDRRFFFTIGLGLLPCSQNKTCQGPNNTMLAASVNNVSFVQPNIALLQSHFLNRSKGVYTTDFPTNPPFKFNYTGTPPSNIMTAKGTKVVVLPFNTSVELVMQDTSIIGAESHPLHLHGFNFVVVGQGFGNFDPKKDPVKFNLVDPAERNTVGVPSGGWVAIRFLADNPGVWFMHCHLEVHTSWGLKMAWVVNDGKRPSQKLPPPPSDLPKC

>PeuLAC7

MLRLLFLLTCALALLASSVASAAIVEHSFYVKNLTVRRLCSEQVVTAVNGSLPGPTLRVREGDTLIVHVFNKSPYNLTIHWHGVFQLLSAWADGPNMVTQCPIPPGGKYTYKFELLQQEGTLWWHAHVSFLRATVYGALVIRPRSGHPYPFPKPHREVPILLGEWWNANVVDVENQAEASGAPPNISDAYTINGLPGDLYNCSQNRMYKLKVQKGKTYLLRIINAALNNQLFFKIANHNMTVVAVDAGYTVPYVTDVVVTGPGQTVDVLLAADQEVGSYFMAANAYASAGPAPPAFPAPPPFDNTTTRGIVVYEGAPTSATPIMPLMPAFNDTPTAHKFFTSISGLAGGPHWVPVPRQIDEHMFVTVGLGLSICPTCLNGTRFSASMNNFSFARPSSLSMLQAFFFNVSEIYTPDFPDNPPVKFDYTNIINAVNPSLLITPKSTSVKVLKYNATVEMVLQNTALLGVENHPMHLHGFNFHVLAQGFGNYDPVNDPKKFNLINPQSRNTINVPVGGWGVIRFTANNPGVWFFHCHLDVHLPFGLATAFVVENGPTSESSLPPPPVDLPQC

>PeuLAC8

MLRLLFLLTCALALLASSVASAAIVEHSFYVKNLTVRRLCSEQVVTAVNGSLPGPTLRVRVGDTLIVHVFNKSPYNLTIHWHGVFQLLSAWADGPSMVTQCPIPPGGKYTYKFELLQQEGTLWWHAHVSFLRATVYGALVIRPRSGHPYPFPKPHREVPILLGEWWNANVVDVENQAEASGAPPNISDAYTINGLPGDLYNCSQNRMYKLKVQKGKTYLLRIINAALNNQLFFKIANHNMTVVAVDAGYTVPYVTDVVVTGPGQTVDVLLAADQEVGSYFMAANAYASAGPAPPAFPAPPPFDNTTTRGIVVYDGAPTSATPIMPLMPAFNDTPTAHKFFTSITGLAGGPHWVPVPRQIDEHMFVTVGLGLSICPTCLNGTRFSASMNNFSFARPSSLSMLQAFFFNVSEIYTPDFPDNPPVKFDYTNIINAVNPSLLITPKSTSVKVLKYNATVEMVLQNTAILGVENHPMHLHGFNFHVLAQGFGNYDPVNDPKKFNLINPQSRNTINVPVGGWGVIRFTANNPGVWFFHCHLDVHLPFGLATAFVVENGPTSESSLPPPPVDLPQC

>PeuLAC9

MLRLLFLLTCALALLTSSVASAAIVEHSFHVKNLTVRRLCSEQVVTAVNGSLPGPTLRVREGDTLIVHVFNKSPYNLTIHWHGVFQLLSAWADGPNMVTQCPIPPGGKYTYKFELLQQEGTLWWHAHVSFLRATVYGALVIRPRSGHRYPFPKPYREVPILLGEWWNANVVDVENQAEAIGAPPNISDAYTINGLPGDLYNCSQNRMYKLKVQKGKTYLLRIINAALNNQLFFKIANHNMTVVAVDAGYTVPYITDVVVTGPGQTVDVLLAADQEVGSYFMAANAYASAGPAPPAFPAPPPFDNTTTRGIVVYEGAPTSATPIMPLLPEFNDTPTAHKFFTSITGLAGGPHWVPVPRQIDEHMFVTVGLGLSICPTCLNRTRLSASMNNFSFARPTSLSMLQAFFFNVSGIYTPDFPDTPPVIFDYTNIINAVNPSLLITPKSTSVKVLKYNAAVEMVLQNTALLGVENHPIHLHGFNFHVLAQGFGNYDPVNDPKKFNLINPQSRNTINVPVGGWGVIRFTANNPGVWFFHCHLDVHLPFGLATAFVVENGPTPESTLPPPPVDLPQC

>PeuLAC10

MLRLLFLLTCALALLASSVASAAIVEHSFYVKNRTVRRLCSEQVVTAVNGSLPGPTLRVREGDTLIVHVFNKSPYDMSIHWHGVFQLLSAWADGPSMVTQCPITPGGKYTYKFKLLQQEGTLWWHAHFSLLRATVYGALIIRPRSGHPYPFPKPNKEVPILLGEWWNANVADIERQAAATGALPNISDAYTINGLPGDLYNCSRNRMYKLKVQKGKTYLLRIINAALDNQLFFKIANHKMTVVAVDAGYTVPYVTDVVVTGPGQTVDVLLAADQEVGSYFMAANAYASAGPAPPAFPAPPPFDNTTTRGIVVYEGAPTSATPIMPLLPEFNDTPTAHKFFTSITGLAGGPHWVPVPRQIDEHMFVTVGLGLSICPTCLNGTRFSASMNNFSFARPSSLSMLQAFFFNVSGIYTPDFPDTPPVIFDYTNIINAVNPSLLITPKSTSVKVLKYNATVEMVLQNTALLGVENHPMHLHGFNFHVLAQGFGNYDPVNDPKKFNLINPVSRNTINVPMGGWGVIRFTANNPGVWFIHCHLEAHLPTGLATALVVENGPTPESTLPPPPVDLPQC

>PeuLAC11

MEIALWIRVLVLVACLFPASVESMVRHYKFNVVMKNSTKLCSTKPIVTVNGQFPGPTLVAREDDTVLVKVVNHVKYNVSIHWHGIRQLRTGWADGPAYITQCPIQPGQSYVYNFTITGQRGTLFWHAHILWLRATVHGAIVILPKRGVPYPFPTPRKEKVIVLGEWWKSDVEAVINEATKSGMAPNVSDAHTINGHPGPVSACSSHGGYNLTVHPGKTYMFRIINAALNEELFFKIAGHQLTVVEVDAAYVKPFKIDTVVIAPGQTTNVLLTANRGSGKYLVAASPFMDAPIAVDNVTATAILHYSGTLASTITTLTVPPAKNATPVATNFTNALRSLNSIKYPARVPLKIDRSLFFTVGLGVNPCATCINGSRVVADINNVTFVMPTIALLQAHVFNISGVFTDDFPANPPTPFNYTGTQPTNFQTVNGTKLYRLAYNNTVQLVLQDTGMLTPENHPIHLHGFNFFEVGRGVGNFDPNKDPKKFNLVDPVERNTIGVPAGGWTAIRFIADNPGVWFMHCHLEVHTTWGLKMAFVVDNGKGPNESVLPPPPDLPKC

>PeuLAC12

MDMALWLRVLVLVACLFPASVESMVRHYKFNVVMKNTTRLCSEKPIVTVNGRFPGPTLVAREDDTVLVKVVNHVKYNVSIHWHGVRQLRTGWADGPAYITQCPIQPRQSFVYNFTITGQRGTLFWHAHILWLRATVHGAIVILPKRGVPYPFPTPHREEVIVLGEWWKSDVEAVINEAMSSGMAPNVSDAHTINGHPGPVSACSSQGGYNLPVQPGKTYLLRIINAALNEELFFKIAGHQLTVVEVDATYVKPFKIDTIVIAPGQTTNVLVTANRGSGRYLVAASPFMDAPIAVDNVTATATLHYSGTLASSATTLTVPPAQNATPVATNFTDALRSLNSIKYPARVPLKIDHSLFFTVGLGVNPCATCVNGSRVVADFNNVTFVMPTIALLQAHFFNIKGVFTDDFPGNPPTPFNYTGTQPTNFQTVNGTKLYRLAYNSTVQLVLQDTGMLTPENHPIHLHGFNFFEVGRGIGNFNPKRDPKKFNLVDPVERNTIGVPAGGWTAIRFIADNPGVWFMHCHLEVHTTWGLKMAFVVDNGKGPNESVLPPPPDLPKC

>PeuLAC13

VKNRTVRRLCSEQVVTAVNGSLPGPTLRVREGDTLIVHVFNKSPYDMSIHWHGVFQLLSAWADGPSMVTQCPITPGGKYTYKFKLLQQEGTLWWHAHFSLLRATVYGALIIRPRSGHPYPFPKPNKEVPILLGEWWNANVADIERQAAATGALPNISDAYTINGLPGDLYNCSRNRMYKLKVQKGKTYLLRIINAALDNQLFFKIANHKMTVVAVDAGYTVPYVTDVVVTGPGQTVDVLLAADQEVGSYFMAANAYASAGPAPPAFPAPPPFDNTTTRGIVVYEGAPASATPIMPLMPAFNDTPTAHKFFTSISGLAGGPHWVPVPRQIDEHMFVTVGLGLSICPTCLNGTRLSASMNNFSFERPSSLSMLQAFFFNVSGIYTPDFPDTPPIKFDYTNIINAVNPSLLITPKSTSVKVMKYNATVEMVLQNTALLGVENHPMHLHGFNFHVLAQGFGNYDPVNDPKKFNLINPVSRNTINVPVGGWGVIRFTANNPGVWFIHCHLEAHLPTGLATALVVENGPTPESTLPPPPVDLPQC

>PeuLAC14

MENYRARAILLLVISIFPALVQCKVRRYDFRVVLTNTTKLCSTKSIVTINGKFPGPTIYAREGDNVNIKLTNHVQYNVTIHWHGVRQLRTGWSDGPAYITQCPIRPGQSYLYNFTLTGQRGTLLWHAHISWLRATIHGAIVILPQKGVPYPFPKPDKEKIIILGEWWKADVEAVVNQATQTGLPPNISDAHIVNGQTGAVPGCPSPGFTLHVESGKTYLLRIINAALNDELFFKIAGHNITVVEVDAAYTKPLSTDTIFIGPGQTTNALLTADKRVGKYLMAVSPFMDTVVAVDNVTAIAFLRYKGTIAFSPPVLTTTPAINATPVTSTFMDNLRSLNSKKYPANVPLTVDHSLYFTIGVGIDPCATCVNGSKAVGAINNISFIMPTTALLQAHYYSISGVFTDDFPAMPPNSFNYTGNNTALNLQTNNGTRIYRLAFNSTVQLVLQGTTIIAPESHPFHLHGFNFFVVGKGFGNFDADNDPKKFNLADPVERNTISVPTAGWAAIRFRADNPGVWFLHCHLEVHTTWGLKMVFVVDNGEGPDESLLPPPSDLPNC

>PeuLAC15

MEYYQARTMLLVIFIFPALVECKVRLYNFRVVLTNTTKLCSTKSIPTINGKFPGPTIYAREGDNVNIRLTNQVQHNVTVHWHGVRQLRTGWADGPAYITQCPILPGQSYLYNFTLTGQRGTLLWHAHISWLRATIHGAIVIFPKKGVPYPFPKPDKEKIITLSEWWKADVEAVINQATMTGLPPNISDAHTVNGQTGAVPGCTSPGFTLHVESGKTYLLRIINAALNDELFFKIAGHNITVVEVDATYTKPFSTDTIFIGPGQTTNALLTADKSIGKYLIAVSPFMDTVVAVDNVTAIAFLRYKGTLAFSPPVLTTTPAINATPATSTFMDKLRSLNSKKYPANVPLTVDHDLYFTIGVGIDPCATCINGSKAVADINNVSFIMPTTALLQAHYYNISGVFTDDFPAKPPITFNYTGNNTAMNLKTTNGTRAYRLAFNSAVQVVLQGTTIIAPESHPFHLHGFNFFVVGKGLGNFDPDNDPKKFNLVDPVERNTVSVPTAGWIAIRFKADNPGVWFLHCHLEVHTTWGLKMAFVVDNGKGPNESIPPPPSDLPSC

>PeuLAC16

MAAALSKKLCWASYILYLYFIYYPAEAAVKRYQFDIQVKNVSRLCHAKPIVTVNGRFPGPTVYAREGDRVLVNVTNHAKYNMSIHWHGLKQFRNGWADGPAYITQCPIKTGHSYTYDFNVTGQRGTLWWHAHILWLRATVYGAIVIMPKPGTPFPFPQPHREEIIIFGEWWKNDVEDIEKQGNKLGLPPNASDAHTINGKPGPLFPCSEKHTFTLEVEQAKTYLLRIINAALNDEFFFAIAGHNMTVVEIDAVYTKPFTTQAIQIAPGQTTNVLVQAAQTPNRYFMAARPFMDAPLSIDNKTATAILQYKGIPNTVLPLLPQLPEPNDTAFALSYNAKLRSLNSPKFQANVPLIVDRHLFYTIGLGINPCPTCLNGTKLTASLNNITFVMPQIGLLQAHYFNIKGVFRLDFPDNPPTPFNYTGAPLTANLVTTLGTRVSKIAYNSTVQLVLQDTNLLTVESHPFHLHGYNFFVVGTGIGNFDPKRDPAKFNLVDPPERNTVGVPTGGWTAIRFRADNPGVWFMHCHLELHTGWGLKTAFVVENGKLPDQSILPPPKDLPPC

>PeuLAC17

MGISFLPSPAFLGLLLFSFVTFSLHPKSAVAITRHYKLDVMLQNVTRLCHTKSMVTVNGKFPGPRIVAREGDRLLIKVVNHVQNNISIHWHGIRQLRSGWADGPAYITQCPIQTGQSYVYNYTIVGQRGTLWWHAHISWLRSTLHGPLIILPKLGTPYPFVKPYKEVPVIFGEWFNADPEAIISQALQTGGGPNVSDAYTINGLPGPLYNCSDKDTFKLRVKPGKTYLLRLINAALNDELFFSIANHTFTVVEADAVYVKPFDTKTLLIAPGQTTNVLLKTKPHRPNAKFFMTARPYVTGQGTFDNTTIAGILEYKESHKTIQSSHSTKKLPLFKPNLPPLNDTSFATNFTRKLRSLANAQFPANVPQKIDRQFFFTVGLGTHSCPQNQTCQGPNGTMFAASVNNVSFAMPTTALLQAHHFGQSKGVYTPDFPINPLTPFNYTGNPPNNTIVSNGTKLVVLPFNTTVELIMQDTSILGAESHPLHLHGFNFFVVGQGFGNFDPNKDPANFNLVDPIERNTVGVPSGGWVAIRFLADNPGVWFMHCHLEVHTSWGLKMAWIVLDGKLPNQKLLPPPADLPRC

>PeuLAC18

MGAPVPASPEILLTILLFAMSCLWAFPDVAGAKHAGITRHYKFNIELTNVTRLCHTKSMVTVNGKFPGPRVVAREGDRLVVKVVNHVPNNITIHWHGIRQLQSGWADGPEYITQCPIQTNQTYVHNFTVTGQRGTLFWHAHLSWLRASVYGPLIIFPKRNVPYPFAKPHKEVTIMLGEWFNADPEAVIRQALQTGGGPNVSEAYTFNGLPGPLYNCSANHTYKLKVKPGKTYLLRLINAALNDELFFSIANHTFTVVEVDATYVKPFETNLLVITPGQTTNVLLKTKPIAPNASFYMLARPYFTGQGTFDNTTVAGILEYETSSNSTAFKPTLPPINATNAVANFTRKLRSLANSQFPVNVPQTVDKTFFFTVGLGNSPCPKNQTCQGPNGTKFAASVNNISMALPSTALLQSYFFKKSNGVFTSDFPSSPLHPFNYTGTPPNNTFVANGTKLVVLPFNTSVEVVMQGTSILGAESHPLHIHGFNFYVVGEGFGNFDPNNDPKNFNLVDPVERNTVGVPSGGWVAIRFHADNPGVWFMHCHFDVHLSWGLRMAWIVLDGTLPSQKLPPPPSDLPKC

>PeuLAC19

MEYSWFRFMLLAVCLFPALVECRIRHYKFNVVMKNTTRLCSSKPIVTVNGLFPGPTLYAREDDTVLVKVVNRVKYNLSIHWHGIRQLRTGWADGPANITQCPIQTGQSYVYNFTITGQRGTLLWHAHILWLRATVHGAIVVLPKLGVPYPFPAPHKEVVVVLAEWWKSDTEAVINEALKSGLAPNVSDAHTINGHPGAVSTCSSQGGFTLPVQSGKTYMLRLINAALNEELFFKIAGHKLTLVEVDATYVKPFKTSTVLIAPGQTTNVLVTTNKNTGKYLVAASPFMDAPIAVDNMTATATLHYSGALSNSPTTLTVPPPKNATAIANQFTNSLRSLNSKTFPAKVPLTVDHSLFFTVGLGINPCPTCKAGNGSRVVASINNVTFVMPTTALLQAHFFNISGVFTTDFPAKPPHVFNYTGTPPTNLQTTSGTKAYRLPYNSTVQLVMQDTGIISPENHPIHLHGFNFFAVGGGVGNYNPKTDPKKFNLVDPVERNTIGVPSGGWVAIRFRADNPGVWFMHCHLEVHTTWGLKMAFLVDNGKGPNESLLPPPSDLPKC

>PeuLAC20

MGASVPASPEILLTILLFAMSCLWAFPEVAGAKHAGITRHYKFNIKLTNVTRLCHTKSMVTVNGKFPGPRVVAREGDRLVVKVVNHVPNNISIHWHGIRQLQSGWADGPEYITQCPIQTNQTYVHNFTVTGQRGTLFWHAHLSWLRASVYGPLIIFPKRNVSYPFAKPHKEVTIMLGEWFNADTEAVISQALQTGGGPNVSEAYTFNGLPGPLYNCSENKTYKLKVKPGKTYLLRLINAALNDDLFFSIANHTFTVVEVDATYAKPFETNLLVITPGQTTNVLLKTKPIAPNASFYMLARPYFTGQGTFDNTTVAGILEYETSSNSTAFKPTLPPINGTNFVANFTRKLRSLANSRFPVNVPQTVDKKFFFTVGLGNSPCPKNQTCQGPNGTKFAASVNNISMALPSSALLQSYFFKKSNGVFTSDFPSSPLHPFNYTGTPPNNTFVANGTKLVVVPFNTSVEVVMQGTRIFGAESHPLHLHGFNFYVVGEGFGNFDPNNDPKNFNLVDPVERNTVGVPTAGWVAIRFHADNPGVWFMHCHFDVHLSWGLRMAWIVLDGTLPSQKLPPPPSDLPKC

>PeuLAC21

MSLLHGVRQLRNPWADGPDRVTQCPIQPGRSYTYRFTIENQEGTLWWHAHSSWLRATVYGALIIHPKLGSPYSFPMPRREIPILLGEWWDRNPMDVLRLADFTGAAPNVSDAYTINGQPGDLYRCSKQETVRFPVDPGETILLRVINSGLNQELFFAVANHILTVVAVDADCTKPFATSFIMIAPGQTTDVLLTADQTPGHYYMAAHAYNSANAPFDNTTTTAILEYKSAPCNANKGKSSTPIFPQLPGFNDTSSAIAFTSSLRSPSKVNVPLQIDENLFFTVGLGLINCTNPNSPRCQGPNGTRFAASINNVSFVLPTRNSLMQAYYQGQPGVFTTDFPPVPPVKFDYTGNVSRGLWQPVKATKLYKLKFGAKVQIVFQDTSIVTVEDHPMHLHGHHFAVVGSGFGNFNPQTDPAKFNLIDPPYRNTIGNPPGGWVAIRLVADNPGIWLLHCHLDSHLNWGLAMAFLVENGGGELQSVQPPPLDLPQC

>PeuLAC22

MGASLLPPPAFLAVFLFSFVTLSVNPEPALAVSKHYKFDVMLQNVTRLCHTKSMVTVNGKFPGPRIVAREGDRLVIRVVNHVQNNISIHWHGIRQLRSGWADGPAYVTQCPIQTGQSYVYNYTIVGQRGTLWWHAHISWLRSTLYGPIILLPKLGTTYPFAKPYKEVPIIFGEWFNADPEAIINQAMQTGGGPNVSDAYTINGLPGPLYNCSAKDTFKLKVKLGKTYLLRMINAALNDELFFSIANHTVTVVDVDAVYVKPFDAETLLITPGQTTNVLLKTKHDYPNAQFFMSARPYATGQGTFDNSTVAGILEYEVPNKTSQSNHSTKKLPLYKPNLPPLNDTSFATNFSSKLRSLASADYPANVPQKVDRQFVFTVGLGTNPCSKNQTCQGPNGTRFAASVNNVSFVMPTTALLQAHHFGQSKGVYSPYFPVSPLIPFNYTGTPPNNTMVSNGTKLVVLPFNTSVELIMQDTSILGAESHPLHLHGFNFFVVGQSFGNFDPSKDPANFNLVDPVERNTVGVPSGGWVAIRFLADNPGVWFMHCHLEVHTSWGLKMAWVVLDGKLPNQKLLPPPADLPKC

>PeuLAC23

MSLLHGVRQLRNPWADGPDRVTQCPIRPGRSYTYRFTIENQEGTLWWHAHSSWLRATVYGALIIHPKLGSPYSFPMPRREIPILLGEWWDRNPMDVLRLADFTGAAPNVSDAYTINGQPGDLYRCSKQETVRFPVDPGETILLRVINSGLNQEFFFAVANHILTVVSVDADFTKPFATSFIMIAPGQTTDVLLTADQTPGHYYMAAHAYNSANAPFDNTTTTAILEYKSAPCNANKGRSSTPIFPQLPGFNDTNSAIAFTSSLRSPSKVNVPLQIDENLFFTVGLGLINCTNPNSPRCQGPNGTRFAASINNVSFVLPTRNSLMQAYYQGQPGVFTTDFPPVPPVKFDYTGNVSRGLWQPVKATKLYKLKFGAKVQIVFQDTSIVTVENHPMHLHGHHFAVVGSGFGNFNPQTDPAKFNLIDPPYRNTIGNPPGGWVAIRLVADNPGIWLLHCHLDSHLNWGLAMAFLVENGVGELQSVQPPPLDLPQC

>PeuLAC24

MDEDNKLESKKETNSNREIATERSRSQQVWSTPNGSSVICFLLSASSPLWSSAGFGITSSILCSRKPIVTVNGRFPGPTLYAREDDTVLVKVVNHVKYNVSIHWHGIRQLRTGWADGPAYITQCPIQPGQNYVYNFTITGQRGTLLWHAHILWLRSTVHGAIVVLPKRGIPYPFPAPHKEVVVVLAEWWKSDTEAVINEALKSGLAPNVSDAHTINGHPGAVSACSSQGGFTLPVRSGETYMLRLINAALNEELFFKIAGHKLTVVEVDATYVKPFKTDTVLIAPGQTTNVLVTTNKNTGNYLVAASPFMDSPIAVDNMTATATLQYSGALANSPTTLTTPPPKNATAVANQFINSLRSLNSRKFPAKVPMNVDHNLFFTVGLGVNPCPSCKAGNGSRVVASINNVTFVMPTTALLQAHFFNISGVFTTDFPAKPPHVFNYSGTPPTNLQTKSGTKVYRLSYNSTVQLVMQDTGIISPENHPIHLHGFNFFAVGRGVGNYNPKTDTKKFNLVDPVERNTIGVPSGGWVAIRFRADNPGVWFMHCHLEVHTTWGLKMAFLVDNGKGPKESLLPPPSDLPKC

>PeuLAC25

MGNSPRPTVLPSMAALQLFCFFIFSLVPDFAAAITRQYTFNITYKNFTRLCHTRSLVTVNGKFPGPRLVAREGDQVLVKVVNHVAENITIHWHGVRQLTSGWADGPAYITQCPIQTGQAYTYNFTITGQRGTLLWHAHISWLRSSLYGPIIILPKLNESYPFKKPYKEIPILFGEWFNVDPEAVIAQALQTGAGPNVSDAYTINGLPGPLYNCSAKDTYKLKVKPGKTYLLRLINAALNDELFFSIANHTLTVVEADAVYVKPFEADTLLITPGQTTNVLLKTKPHLPNATFYMFAGPYFSGMGSFDNSTTAGFLVYKHPSGNNHLKKLPSLKPTLPPTNATGFVANFTKKFRSLANAKFPANVPQTVDRKFFFTVGLGTHPCPKNTTCQGPNNNTKFAASINNVSFVLPSVALLQSYFFGQSNGVFTSDFPQNPTIPFNYTGTPPNNTMVSNGTKTVVLAFNTSVELVMQGTSILGAESHPLHLHGYNFFVVGQGFGNYDPNKDPSNFNLVDPVERNTAGVPAGGWVAIRFLADNPGLWFMHCHLDVHTSWGLRMAWIVLDGPQPNQKIPPPPSDLPKC

>PeuLAC26

MGASFLPSPAFLAVFLISFVTLSIHPEPALAITRHYKFDVMLQNVTRLCHTKSIVTVNGKFPGPRIVAREGDRLIIKVVNHVQNNISIHWHGIRQLRSGWADGPAYITQCPIQTGQSYVYNYTTVGQRGTLWWHAHISWLRSTLHGPLIILPKLGTTYPFAKPHKEVPIIFGEWFNADPEAIITQAMQTGGGPNVSDAYTINGFPGPLYNCSAKDTFKLKVKPGKTYLLRMINAALNDELFFSIANHTLTVVDVDAIYVKPFDTETLLIAPGQTTNVLLKTKPHHPNASFFMSARPYVTGQGTFDNSTVAGILEYEESNKTIKSSHSPKKLPLYKPNLPPLTDTSFATNFTSKLRSLASAEFPANVPRKVDRQFFFSVGLGTNPCSKNQTCQGPNGTMFAASVNNVSFVMPTKALLQAHHFGQSKGVYSPNFPINPPIPFNYTGTPPNNTMVNNGTKLVVLPFNASVELIMQDTSILGAESHPLHLHGFNFFVVGQGFGNFDAKKDPANFNLVDPVERNTVGVPSGGWVAIRFLADNPGVWFMHCHLEVHTSWGLKMAWVVLDGKLPNQKLLPPPADLPKC

>PeuLAC27

MEGGRKHYGILLEALAIIAAAVPCCSSQTIRRFQFNVEWKQVTRLCTTKQLLMVNGQYPGPTIAVHEGDNVEIKVKNRIAQNTTLHWHGVRQLRTGWADGPAYVTQCPIRGGQSYTYKFTVTGQRGTLLWHAHYAWQRASVYGAFIIYPRIPYPFSHQIQAEIPIIFGEWWNADPAEVENTMMLTGAGPDSSNAYTINGLPGPLYPCSNQDTFIQTVEYGKTYMLRIINAALADELFFAIAKHTLTVVEVDAVYTKPFATTSIMIAPGQTTTVLMTANQVPDFTGMFVMAARPYLTSVFPFNNSTTIGFLRYKNARTWKGKSPVDPSSLKLPNLPEMEDTAFATKFSDKIKSLASPQHPCNVPKTIDKRVITTISLNIQDCPENKTCLGYKGKSFFASMNNQSFVRPSISILESYYKNLTTSSFSSDFPEKPPNYFDYTGGDPLTQNMNTKFGTKLLVLPYGTNVEIVLQDTSFLNLENHPIHVHGHNFFIVGSGFGNFNKAKDPKRYNLVDPPERNTVAVPSGGWAAIRIKADNPGVWFVHCHLEQHTSWGLATGFIVQNGQGPSQSILPPPQDLPSC

>PeuLAC28

MEGVHKHYGILLASLAIIAAALPCCSSQTTRRFQFNVEWKQVTRLCTTKQLLMVNGQYPGPTIAVHEGDNVEINVKNRIAQNTTLHWHGVRQLRTGWADGPAYVTQCPIRGGQSYTYKFTVTGQRGTLLWHAHYAWQRASVYGAFIIYPRIPYPFSHQIQAEIPIIFGEWWNADPDEVENVMMITGAGPDSSNAYTINGLPGPLYPCSNQDTFIQTVEYGKTYMLRIINAALADELFFAIAKHTLTVVEVDAVYTKPFATTSIMIAPGQTTTVLMTANQVPDFTGMFVMAARPYLTSVFPFNNSTTIGFLRYKNARTWKGKSPVDPSSLKLPNLPEMEDTAFATKFSDKIKSLASPQHPCNVPKTIDKRVITTISLNIQDCPENKTCLGYKGKSFFASMNNQSFVRPSISILESYYKNLTTSSFSSDFPEKPPNNFDYTGGDPLTQNMNTKFGTKLIVVPYGTNIEVVLQDTSLVNLENHPIHVHGHNFFIVGSGFGNFNEAKDPKRYNLVDPPERNTVAVPSGGWAAIRIKADNPGVWFIHCHLEQHTSWGLATGFIVQNGQGPSQSMLPPPQDLPSC

>PeuLAC29

MATESSEGEEEGKITGGNKLLVIEDDLREMGKKAAWSVSSCKPGNGVSFLRDDNLDTYWQSDGAQPHFVNIQFQKKVKLQLVVLYVDFKLDESYTPSKISIRAGDGFHNLKEIKTMELVKPTGWVYLSLSGNDPRETFVNTFMLQIAVLSNHLNGRDTHVRQIKVHGPRLHGLKQYRNGWADGPAYITQCPIQTGSSYTHDFNVTGQRGTLWWHAHILWLRATVYGAIVIMPKQGTRYPFPQPNMEVPILLGEWWNADVEEIEKQGTEMGLPPNMSDAHTINGKPGPLFPCSEKHTFAMEIESGKTYLLRIINAALNDELFFGIAGHNMTVVEVDGVYTKPFSIQSLLIAPGQTTNVLVQANQVPGRYFMATRAFMDVPLPVDNKTATAILQYKGIPNTVLPSLPQLPASNDTEFALGYNRKLKSLNTPQFPANVPLKVDRNLFYTVGFGKDPCPTCVNGTRLLASLNNISFVMPQIGLLQAHYFNISGVFKTNFPDKPPTPFNYTGAPLTASLGTVHGTRLSKIAFNSTVELVLQDTNLLTVESHPFHLHGYNFFVVGTGIGNFDPAKDPAKYNLVDPVERNTVGVPAGGWTAIRFRADNPGVWFMHCHLELHTGWGLKTAFVVEEGPGSDQSILPPPKDLPPC

>PeuLAC30

MQASLRDQMEVIKSIFADRHCSFFLVVMLLASTLSLAIAEIHHHDFVLTNHSNSTILWHIVYLAIDLRSSYFFRHGVRQIRTGWADGPEFVTQCPIRPGGSYTYRFTIEGQEGTLWWHAHSSWLRATVYGAIIILPREGSSYPFTKPKRETPILLGEWWDANPVDVVREATRTGAAPNISDAYTINGQPGDLYNCSSEDTTIVPIASGETNLLRVINAALNQPLFFTIANHKFTVIGADASYLKPFTTSVIMLGPGQTTDVLISGDQLPGRYYIAARAYQSTQNAPFDNTTATAILEYKSALCPAKCTTKPVMPRLPAYNDTATVTAFTRSLRSPRKVDVPTDIDENLFFTIGLGLNNCPKNFRARRCQGPNGTRFTASINNVSFVFPSNIALLQAYRQKVPGIYTTDFPAKPPVKFDYTGNVSRSLFQPVRGTKLYKLKYGSRVQIVLQDTSIVTPENHPMHLHGYDFYVIAEGFGNFNPKTHKSKFNLVDPPMRNTVAVPTNGWAVIRFVADNPGVWIMHCHLDVHITWGLAMAFLVEDGIGELQSVEPPPADLPIC

>PeuLAC31

MAYVNLPLQTAGVAMEGGHKNCGILLVSLVIIAGAVPFCSSQTTRRFQLNVEWKKVTRLCTTKQLLTVNGQYPGPTIAVHEGDRVEIKVKNRIAHNTTLHWHGLRQLRTGWADGPAYITQCPIRGGQSYTYKFSVINQRGTLLWHAHYAWQRASVYGALIIYPRMPYPFLAQIQAEIPIILGEWWNGDPDEVEKIMMLTGAGPDSSDAYTINGMPGPLYPCSNRDTFIKTVEYGRTYMLRIINAALTNELFFAIAKHKLTVVEVDAVYTKPFTTTSIMIAPGQTTTVLMTANQVPDSTGMFAMAARPYLTSVFPLNNSTTISFLRYKNARNRRGKPPFNPSSLKLYNLPAMEDTTLATKFSGNIKSLASPEYPCNVPKTIDKRVITTISLNLQDCPAKKTCLGFRGKKFFASMNNQSFVRPSISILESYYKNLTTTSFSSDFPEKPPNAFDYTGGDPLSQNMNTEFGTKLIVVPYGTNLEIVLQDTSFLNLENHPIHVHGHNFFIVGSGFGNFNKAKDPKRYNLVDPPERNTVAVPSGGWAAIRIKADNPGVWFMHCHLEQHTSWGLAAGFIVQNGQEPSQRLLPPPQDLPSC

>PeuLAC32

MEVINRIFASRHCSFFLLLLLLASAMHGIRQMRTGWADGPEYVTQCPIRPGRSYTYRFTIEGQEGTLWWHAHSSWLRATVYGALIIHPREGSSYPFSKPNRETPILLGEWWDTNPIDVVREATRTGAAPNISDAYTINGQPGDLYNCSSKDTTIVPIDSGETNLLRVINAALNQPLFFTIANHKFTVVGADASYVKPFTTSVIMLGPGQTTDVLISGDQLPGRYYMAARAYQSAQNAPFDNTTTTAILEYKSVLCPAKCTKKPFMPPLPAYNDTATVTAFSRSFRSPRKVDVPTDIDENLFFTIGLGLNNCPKNFRARRCQGPNGTRFTASMNNVSFVFPSKASLLQAYKQKIPGVFTTDFPANPQVKFDYTGNVSRSLFQPLPGTKLYKLKYGSRVQVVLQDTSIVTPENHPIHLHGYDFYVIAEGFGNFNHKTDKSKFNLVDPPMRNTVAVPVNGWAVIRFVADNPGVWIMHCHLDVHITWGLAMAFLVEEGIGILQSVEPPPADLPIC

>PeuLAC33

MPKVTTMILLHGVRQMRTGWADGPEFVTQCPIRPGGSYTYRFTIQGQEGTLWWHAHSSWLRATVYGALIIHPKEGSSYPFSKQPKRETAILLGEWWNANPIDVVREATRTGGAPNSSDAYTINGQPGDLYNCSSQDTVIVPIDSGETNLLRVINAALNQPLFFTVANHKLTVVGADASYVKPFTTSVLMLGPGQTTDVLISGDQNPSRYYMAARAYQSAQNAPFDNTTTTAILEYKSSLCAAKNCSSNKPIMPPLPSFNDTATVTAFTSSFKSTDKTFVPTDIDESLFFTVGLGLNPCPPNFNKSRQCQGPNGTRFTASMNNVSFVLPSNFSLLQAHHQRIQGVFTTDFPANPPRKFDYTGNVSRSLFTPVPGTKLYRLKYGSRVQIVLQDTSIVTSENHPIHLHGYDFYIIAQGFGNYNPRTDPSKFNLVDPPLRNTVGVPVNGWAVIRFVADNPGVWLMHCHLDVHITWGLATAFLVENGVGELQSIESPPEDLPLC

>PeuLAC34

MPKVTTMILLHGVRQMRTGWADGPEFVTQCPIRPGGSYTYRFTIQGQEGTLWWHAHSSWLRATVYGALIIHPKEGSSYPFSKQPKRETAILLGEWWNANPIDVVREATRTGGAPNSSDAYTINGQPGDLYNCSSQDTVIVPIDSGETNLLRVINAALNQPLFFTVANHKLTVVGADASYVKPFTTSVLMLGPGQTTDVLISGDQNPSRYYMAARAYQSAQNAPFDNTTTTAILEYKSSLCAAKNCSSNKPIMPPLPSFNDTATVTAFTSSFKSTDKTFVPTDIDESLFFTVGLGLNPCPPNFNKSRQCQGPNGTRFTASMNNVSFVLPSNFSLLQAHHQRIQGVFTTDFPANPPRKFDYTGNVSRSLFTPVPGTKLYRLKYGSRVQIVLQDTSIVTSENHPIHLHGYDFYIIAQGFGNYNPRTDPSKFNLVDPPLRNTVGVPVNGWAVIRFVADNPGVWLMHCHLDVHITWGLATAFLVENGVGELQSIESPPEDLPLC

>PeuLAC35

MTHGVRQMRTGWADGPEFVTQCPIRPGGSYTYKFTIQGQEGTLWWHAHSSWLRATVYGALIVHPKEGSPYPFSKQPKRETAIVLGEWWNANPIDVVREATRTGGAPNVSDAYTVNGQPGDLYNCSSQDTVIVPIDSGETNLLRVINSALNQPLFFTVANHKLTVVGADASYVKPFTTSVLMLGPGQTTDVLISGDQTPSRYYMAARAYQSAQNAPFDNTTTTAILEYKSSSCAAKNCSANKPIMPPLPAYNDTATVTNFTTSFKSADKNLVPTDIDESLFFTIGLGLNPCPSNFNKSSQCQGPNGTRFTASMNNVSFVLPSNFSLLQAHHQRIQGVFTTDFPANPPRKFDYTGNVSRSLFQPVAGTRLYNLKYGSRVQLVLQDTSIVTPENHPIHLHGYDFYIIAQGFGNYNPRTDPSKFNLVDPPLRNTVAVPVNGWAVIRFVADNPGVWLMHCHLDVHITWGLATAFLVENGVGELQSIESPPEDLPLC

>PeuLAC36

MANLVTSFMLWLCLISYAYTTIHAAPEWPRGRSTRFYDFKIQTTTVDKLCNSKQIVTVNNMFPGPVVYAQQGDKLIVKVSNESPYNATIHWHGVRQILSCWFDGPSYITQCPIQPGQTFTYEFTLVGQKGTFFWHAHVSWLRATVYGALVVYPKPGIPYPFKYPYEEHIVILGEYWLQDIVQLERQVVASGGGPPPANAYTINGHPGPNYNCSANDVYKIDVVPGKTYLLRLINAGLNMENFFAIANHKLTIVEADAEYTKPFTTDRVMLGPGQTMIVLVTADQTIGKYSMAMGPYVSGQNIPFQNISAIAYFQYVGAMPNSLSLPARLPSFNDNLAVKTVMDGLRGLNTSDVPKEIDTNLFLTIGMNVNKCTSKTPQQNCQGLNNGTMAASMNNISFIKPTVSVLEAYYKGIDGFFTDNFPGAPFRFYDFVNGAPNNAPNDTSSMNGTRVKVLEYGTRVQMILQNTGTVTTENHPIHLHGYSFYVVGYGAGNYNPQTVNLNLVDPPYMNTIGVPVGGWAAIRFVADNPGVWFMHCHFDIHQSWGLGTVFIVKNGKGHLETLPHPPADLPRC

>PeuLAC37

MTIHWHGLKQFRNGWADGPAYITQCPIKTGHSYTYDFKVTGQRGTLWWHAHIFWLRATVYGAIVIMPKPGTPFPFPQPHREETIVLGEWWNNDVEETEKQGRKLGLPPNASDAHTINGKPGPLFPCSEKHTFAMEVEQGKTYLLRIINAALNDELFFAIAGHNMTVVEVDAVYTKHFTTQAVLIAPGQTTNVLVQATQSPNRFFMAARPFMDAPLTVDNKTATAILQYKGIPNTVIPILPKLPAPNDTAFALSYNAKLRSLNSPQFPANVPLKVDRHLFYAIGLGINPCPSCLNGTRLTASLNNITFVMPQIGLLQAHYFNTKGVFRLDFPDNPPSPFNYTGVPLTANLGTTLGTRLSKVVYNSTVQLDPAKFNLVDPPERNTVGVPTGGWTAIRFKADNPGVWFMHCHLELHTGWGMKTAFVVENGVGPDQSIMPPPKDLPPC

>PeuLAC38

MGSSWFRFMLLDVSLFPALVECRVRHYKFNVVMKNTTRLCSSKPIVTVNGRFPGPTLHAPEDDTVLVKVINHIKYNVSIHCHGIRQLRTGWADGPANITQCPIQTGQSYVYNFTITGQRGTLLWHAHILWLRATVHGAIVVLPKLGVPYPFPAPHKEFVVVLAEWWKSDTEAVINEALKSGLAPMSLMLPQLMGIQELSQLVLHSLTQECSSILAGGFTLPVESGKTYMLRLINAALNEELFFKIAGHKLTLVEVDATYVKPFKTSTVLIAPGQTTNVLVTTNKNTGKYLVAASPFMDAPIAVDNMTATATLHYSGALSSTPTAPTIDSCNMAATYYLYPYITPTTLTIPPPKNATAVANQFTNSLRTLNSKRFPAKVPLTVDHNLIFTVGLGVNPCSTCKAGNGSRVVASVNNVTFVMPTTALLQAHFFNISGVWFMHCHLDVHTTWGLKMAFMVDNGKGPNESTLPPPNDFPKC

>PeuLAC39

MQASLRDQMEVIKHGVRQIRTGWADGPEFVTQCPIRPGGSYTYRFTIEGQEGTLWWHAHSSWLRATVYGAIIILPREGSSYPFTKPKRETPILLGEWWDANPVDVVREATRTGAAPNISDAYTINGQPGDLYNCSSEDTTIVPIASGETNLLRVINAALNQPLFFTIANHKFTVIGADASYLKPFTTSVIMLGPGQTTDVLISGDQLPGRYYIAARAYQSTQNAPFDNTTATAILEYKSALCPAKCTTKPVMPRLPAYNDTATVTAFTRSLRSPRKVDVPTDIDENLFFTIGLGLNNCPKNFRARRCQGPNGTRFTASINNVSFVFPSNIALLQAYRQKVPGIYTTDFPAKPPVKFDYTGNVSRSLFQPVRGTKLYKLKYGSRVQIVLQDTSIVTPENHPMHLHGYDFYVIAEGFGNFNPKTHKSKFNLVDPPMRNTVAVPTNGWAVIRFVADNPGVWIMHCHLDVHITWGLAMAFLVEDGIGELQSVEPPPADLPIC

>PeuLAC40

MDSPIAVDNMTATATLQYSGALANSPTTLTTPPPKNATAVANQFINSLRSLNSRKFPAKVPLNVDHNLFFTVGLGVNPCPSCKAGNGSRVVASINNVTFVMPTTALLQAHFFNISGVFTTDFPAKPPHVFNYTGTPPTNLQTKSGTKVYRLSYNSTVQLVMQDTGIISPENHPIHLHGFNFFAVGRGVGNYNPKTDTKKFNLVDPVERNTIGVPSGGWVAIRFRADNPGVWFMHCHLEVHTTWGLKMAFLVDNGKGPKESLLPPPSDLPKC
